# Supplementary material for: Assessing public support for extending smoke-free policies beyond enclosed public places and workplaces: protocol for a systematic review and meta-analysis
Source: BMJ Open. 2021 Feb 5;11(2):e040167. doi: 10.1136/bmjopen-2020-040167 (PMC7925902; doi:10.1136/bmjopen-2020-040167)
Supplement: Supplementary data [file bmjopen-2020-040167supp001.pdf]

Appendix I

embase.com (1974-)

('smoking regulation'/exp OR (smokefree OR ((smoking OR smoke OR tobacco) NEAR/3 (regulation* OR government* OR law OR laws OR policy OR policies OR ban OR bans OR banned OR free OR restrict* OR act OR acts))):ab,ti) AND ('public opinion'/exp OR 'public attitude'/de OR (opinion* OR support* OR views OR (public NEAR/3 view) OR attitude* OR feeling* OR acceptance* OR accepts OR perception* OR misperception*):ab,ti) NOT ([Letter]/lim OR [Note]/lim OR [Editorial]/lim)

Medline Ovid (1946-)

(Smoke-Free Policy/ OR (smokefree OR ((smoking OR smoke OR tobacco) ADJ3 (regulation* OR government* OR law OR laws OR policy OR policies OR ban OR bans OR banned OR free OR restrict* OR act OR acts))).ab,ti.) AND (Public Opinion/ OR Attitude/ OR (opinion* OR support* OR views OR (public ADJ3 view) OR attitude* OR feeling* OR acceptance* OR accepts OR perception* OR misperception*).ab,ti.) NOT (news OR book* OR chapter* OR dissertation abstract*).pt.

Web of science Core Collection (1975-)

TS=(((smokefree OR ((smoking OR smoke OR tobacco) NEAR/2 (regulation* OR government* OR law OR laws OR policy OR policies OR ban OR bans OR banned OR free OR restrict* OR act OR acts)))) AND ((opinion* OR support* OR views OR (public NEAR/2 view) OR attitude* OR feeling* OR acceptance* OR accepts OR perception* OR misperception*)))

PsycINFO Ovid (1806-)

((smokefree OR ((smoking OR smoke OR tobacco) ADJ3 (regulation* OR government* OR law OR laws OR policy OR policies OR ban OR bans OR banned OR free OR restrict* OR act OR acts))).ab,ti.) AND (Public Opinion/ OR Attitudes/ OR (opinion* OR support* OR views OR (public ADJ3 view) OR attitude* OR feeling* OR acceptance* OR accepts OR perception* OR misperception*).ab,ti.) NOT (news OR book* OR chapter* OR dissertation abstract*).pt.

LILACS

(smokefree OR smoking OR smoke) AND (opinions OR views OR attitudes)

Scientific Electronic Library Online [SciELO])

(smokefree OR smoking OR smoke) AND (opinions OR views OR attitudes)

WHO Global Health Library

(smokefree OR smoking OR smoke) AND (opinions OR views OR attitudes)
