# Supplementary material for: Assessing public support for extending smoke-free policies beyond enclosed public places and workplaces: protocol for a systematic review and meta-analysis
Source: BMJ Open. 2021 Feb 5;11(2):e040167. doi: 10.1136/bmjopen-2020-040167 (PMC7925902; doi:10.1136/bmjopen-2020-040167)
Supplement: Author's manuscript [file bmjopen-2020-040167.draft_revisions.pdf]

BMJ Open

BMJ Open is committed to open peer review. As part of this commitment we make the peer review history of every article we publish publicly available.

When an article is published we post the peer reviewers' comments and the authors' responses online. We also post the versions of the paper that were used during peer review. These are the versions that the peer review comments apply to.

The versions of the paper that follow are the versions that were submitted during the peer review process. They are not the versions of record or the final published versions. They should not be cited or distributed as the published version of this manuscript.

BMJ Open is an open access journal and the full, final, typeset and author-corrected version of record of the manuscript is available on our site with no access controls, subscription charges or pay-per-view fees (http://bmjopen.bmj.com).

If you have any questions on BMJ Open's open peer review process please email info.bmjopen@bmj.com

BMJ Open

Assessing public support for extending smoke-free policies beyond enclosed public places and workplaces: protocol for a systematic review and meta-analysis

Journal:	*BMJ Open*
Manuscript ID	bmjopen-2020-040167
Article Type:	Protocol
Date Submitted by the Author:	06-May-2020
Complete List of Authors:	Boderie, Nienke; Erasmus MC, University Medical Centre Rotterdam, Department of Public Health<br>Mölenberg, Famke; Erasmus MC, University Medical Centre Rotterdam, Department of Public Health<br>Sheikh, Aziz; University of Edinburgh, Centre of Medical Informatics, Usher Institute ; Brigham and Women's Hospital/Harvard Medical School, Division of General Internal Medicine and Primary Care<br>Bramer, Wichor; Erasmus MC, University Medical Centre Rotterdam, Medical Library<br>Burdorf, Alex; Erasmus MC, University Medical Centre Rotterdam, Department of Public Health<br>Van Lenthe, Frank; Erasmus MC, University Medical Centre Rotterdam, Department of Public Health<br>Been, Jasper; Erasmus MC, University Medical Centre Rotterdam, Department of Public Health; Erasmus MC - Sophia Children’s Hospital, University Medical Centre Rotterdam, Department of Paediatrics
Keywords:	PUBLIC HEALTH, PREVENTIVE MEDICINE, Health policy < HEALTH SERVICES ADMINISTRATION & MANAGEMENT

SCHOLARONE™
Manuscripts

I, the Submitting Author has the right to grant and does grant on behalf of all authors of the Work (as defined in the below author licence), an exclusive licence and/or a non-exclusive licence for contributions from authors who are: i) UK Crown employees; ii) where BMJ has agreed a CC-BY licence shall apply, and/or iii) in accordance with the terms applicable for US Federal Government officers or employees acting as part of their official duties; on a worldwide, perpetual, irrevocable, royalty-free basis to BMJ Publishing Group Ltd ("BMJ") its licensees and where the relevant Journal is co-owned by BMJ to the co-owners of the Journal, to publish the Work in this journal and any other BMJ products and to exploit all rights, as set out in our licence.

The Submitting Author accepts and understands that any supply made under these terms is made by BMJ to the Submitting Author unless you are acting as an employee on behalf of your employer or a postgraduate student of an affiliated institution which is paying any applicable article publishing charge ("APC") for Open Access articles. Where the Submitting Author wishes to make the Work available on an Open Access basis (and intends to pay the relevant APC), the terms of reuse of such Open Access shall be governed by a Creative Commons licence – details of these licences and which Creative Commons licence will apply to this Work are set out in our licence referred to above.

Other than as permitted in any relevant BMJ Author's Self Archiving Policies, I confirm this Work has not been accepted for publication elsewhere, is not being considered for publication elsewhere and does not duplicate material already published. I confirm all authors consent to publication of this Work and authorise the granting of this licence.

For peer review only

Assessing public support for extending smoke-free policies beyond enclosed public places and workplaces: protocol for a systematic review and meta-analysis

Registration number …

Nienke W. Boderie[1], Famke J.M. Mölenberg[1], Aziz Sheikh[2,3], Wichor M. Bramer[4], Alex Burdorf[1], Frank J. van Lenthe[1], Jasper V. Been[1,2,5]

[1] Department of Public Health, Erasmus MC, University Medical Centre Rotterdam, Rotterdam, Netherlands
[2] Centre of Medical Informatics, Usher Institute, The University of Edinburgh, Edinburgh, UK
[3] Division of General Internal Medicine and Primary Care, Brigham and Women's Hospital/Harvard Medical School, Boston, MA, USA
[4] Medical Library, Erasmus MC, University Medical Centre Rotterdam, Rotterdam, Netherlands
[5] Department of Paediatrics, division of Neonatology, Erasmus MC - Sophia Children's Hospital, University Medical Centre Rotterdam, Rotterdam, Netherlands

Corresponding author:
Jasper V. Been
Erasmus University Medical Centre, PO Box 2040, 3000 CA Rotterdam
j.been@erasmusmc.nl
+31 10 70 36077

Word count: 3135

For peer review only

KEYWORDS: Smoke-Free Policy, Tobacco Smoke Pollution, Attitude

ABSTRACT: (296 words)

Introduction: Smoke-free enclosed public environments are effective in reducing exposure to second-hand smoke and yield major public health benefits. Building on this, many countries are now implementing smoke-free policies regulating smoking beyond enclosed public places and workplaces. In order to successfully implement such 'novel smoke-free policies' public support is essential. We aim to provide the first comprehensive systematic review assessing levels and determinants of public support for novel smoke-free policies.

Methods and analysis: The primary aim of this review is to summarise the level of public support for novel smoke-free policies. Eight online databases (from 1 January 2004, no language restrictions) will be searched by two independent researchers. Studies are eligible if assessing support for novel smoke-free policies in the general population (age ≥16 years) and have a sample size of n≥400. Studies funded by the tobacco industry or evaluating support among groups with vested interest are excluded. The primary outcome is proportion of public support for smoke-free policies, subdivided according to the spaces covered: (1) indoor private spaces (e.g. cars), (2) indoor semi-private spaces (e.g. multi-unit housing), (3) outdoor (semi-)private spaces (e.g. courtyards), (4) non-hospitality outdoor public spaces (e.g. parks, hospital grounds, playgrounds), and (5) hospitality outdoor public spaces (e.g. restaurant terraces).The secondary aim is to identify determinants associated with public support on three levels: (1) within-study determinants (e.g. smoking status), (2) between-study determinants (e.g. survey year), and (3) context-specific determinants (e.g. social norms). Risk of bias will be assessed using the Mixed Methods Appraisal Tool (MMAT) and a sensitivity analysis will be performed excluding studies at high-risk-of-bias.

Ethics and dissemination: No formal ethical approval is required. Findings will be disseminated to academics, policy-makers and the general public.

The protocol is registered with PROSPERO registration number: x

STRENGHTS AND LIMITATIONS

- This systematic review is unique in providing a structured overview of levels of public support for 'novel smoke-free policies' (i.e. smoke free policies that go beyond regulating smoking in enclosed public places and workplaces)
- Within- and between-study determinants associated with public support will be assessed, and thematic synthesis will be used to identify context-specific determinants.
- The protocol presented has been designed in line with the Preferred Reporting Items for Systematic Reviews and Meta-Analysis for Protocols (PRISMA-P)
- The generalizability and value of this systematic review depends on the availability and quality of the data.

For peer review only

INTRODUCTION

Second-hand smoke (SHS) exposure is related to 1.2 million deaths per year.[1] Smoke-free environments have proven to be effective in reducing exposure to SHS and have major public health benefits.[2] Previous systematic reviews reported consistent evidence for improved cardiovascular health and reduced smoking-related mortality, as well as reductions in preterm birth, severe asthma exacerbations and respiratory tract infections in children, following implementation of smoke-free legislation in indoor public places and workplaces.[3-5]

In 2004, Ireland was the first country in the world to implement comprehensive smoke-free legislation covering enclosed workplaces and public places, and many more countries followed its example.[6] An increasing number of jurisdictions is now implementing, or considering implementing, additional smoke-free policies that go beyond regulating smoking in enclosed public places, henceforth referred to as 'novel smoke-free policies', in an attempt to further improve population health via reducing SHS exposure. For example, several countries have implemented laws requiring private cars carrying children be smoke-free,[7] the city of New York banned smoking in all public parks, pedestrian plazas and at all beaches,[8] and the US Department of Public Housing and Urban Development requires all public housing units to be smoke-free, both within resident units and in public areas.[9] Public support is essential in democracies in order for policy-makers to consider implementing such novel smoke-free policies and to increase the likelihood of successful implementation,[10] and accordingly the World Health Organisation (WHO) stated that "Involving civil society is central to achieving effective legislation".[5]

However, pubic support may vary over time, as well as by population subgroups. For example, women and non-smokers tend to be more in favour of smoke-free legislation than men and current smokers.[11] Several studies showed that public support for smoke-free policies increased after successful implementation and particularly so among smokers.[12-14] Furthermore, public support for smoke-free policies was higher when policies covered spaces that were frequently visited by those more vulnerable to the adverse health effects of SHS.[15] For example, in the US and Canada public support for smoke-free playgrounds (89-91%) was substantially higher than for smoke-free outdoor workplaces (12-46%) and sidewalks (31-49%).[11] Context-specific determinants may also contribute to differences in public support across settings. Aspects that enhanced successful adoption of smoke-free zones at outdoor school grounds at secondary schools included communication about the policy, collaboration between and within stakeholders, social norms, and evidence about the effectiveness of smoke-free zones.[16]

A structured overview of the levels and determinants of public support for smoke-free policies beyond enclosed public places and workplaces across various settings is currently lacking. Having this information available may help governments, policy-makers and advocates to successfully implement or promote smoke-free policies. To address this gap in the literature, we will conduct a systematic review of studies assessing public support for novel smoke-free policies. The primary aim of the review is to summarise the level of public support across the globe for novel smoke-free policies and to evaluate if public support changed following implementation of the novel smoke-free policies. The secondary aim is to identify determinants associated with public support at the following three levels: 1) within-study determinants (e.g. age, smoking status, parental status), 2) between-study determinants (e.g. income level of the country, whether smoke-free legislation in enclosed public places and workplaces was already in place), and 3) context-specific determinants (e.g. setting, framing, enforcement of smoke-free policies).

For peer review only

METHODS AND ANALYSIS

We used the Preferred Reporting Items for Systematic Reviews and Meta-Analysis Protocols (PRISMA-P) guidelines to facilitate development of this protocol, see appendix I. The protocol has been registered in PROSPERO as …

In this review, we will use the term 'traditional smoke-free legislation' to refer to smoke-free legislation covering enclosed public places and workplaces (i.e. compliant with Article 8(2) of the Framework Convention on Tobacco Control; FCTC) and the term 'novel smoke-free policies' to refer to policies and legislation regulating smoking in any other places, such as (semi)private places and (partially) outdoor spaces, whether public or (semi)private.[17] The definition proposed here relates to policies, not necessarily enacted via formal legislation, as this will allow us to evaluate local smoke-free initiatives as well as nation-wide programmes.

Eligibility criteria

We will include articles published in scientific journals as well as 'grey literature' evaluating public support for novel smoke-free policies covering (semi)private places and (partially) outdoor spaces, whether public or (semi)private. Grey literature includes policy documents and reports that are published non-commercially and/or are not indexed by major scientific literature databases. Quantitative studies will be sought with no restrictions regarding language. We will seek translation for reports in foreign languages to assess eligibility. Studies for which only an abstract is available, will not be included since risk of bias for these studies cannot be adequately assessed.

Eligibility of the studies will be assessed using the following criteria:

(1) Studies will be eligible if support for one or more novel smoke-free policies is evaluated. We will include studies assessing support for novel smoke-free policies that are already in place as well as those assessing support for upcoming or theoretical implementation of such policies. Policies at any level are eligible, such as city-level, state-level, country level etc.. Studies will be excluded if solely evaluating traditional smoke-free legislation.[17]

(2) Studies will be eligible if they assessed public support for smoke-free policies in the population aged 16 years or above who represent the majority of a population primarily affected by the policy (e.g. students in case of a smoke-free school policy), or in any of the predefined population subgroups (see *within-study determinants of public support* below). We set this age criterion to include the part of the population that is entitled to vote in most democracies, and as such may be regarded to be of particular interest to politicians and policymakers. Any study reporting (sub)populations in which at least 50% fits this age criterion will also be included.

(3) Studies will be included if the survey sample consisted of a minimum of 400 persons. This sample size was set to infer study estimates back to the target population with a two-sided alpha level of 0.025 and a 5% margin of error.[18] Similar criteria were used by earlier reviews assessing public support.[19]

(4) Studies will be included when published from 1 Jan 2004 onwards. This pragmatic cut-off was chosen as the first national traditional smoke-free law covering indoor public places and workplaces was introduced in Ireland in 2004. Hence, assessments of public support for novel smoke-free policies are unlikely to have preceded 2004, and are unlikely to be relevant for current everyday practice if they have.

For peer review only

(5) Studies will be excluded if solely evaluating support among specific subgroups not representing the majority of the population primarily affected by the policy, policy makers or groups with clearly vested interest, e.g. opinion of tobacco industry groups. Studies funded by the tobacco industry will also be excluded.

Information sources

The following electronic databases will be searched for eligible studies: (1) embase.com, (2) Medline ALL Ovid, (3) Web of Science Core Collection, (4) WHO Library Database (WHOLIS), (5) Latin American and Caribbean Health Sciences Literature (LILACS), (6) Scientific Online Library Online (SciELO), (7) PsychINFO and (8) Google Scholar.

Search strategy

The specific search strategies per database have been created in close collaboration with a bibliographic expert of the Erasmus MC with expertise in systematic reviewing (WMB; see Appendix 1). Search terms include three parts: (1) terms to identify smoke-free policies; (2) terms to identify measures of public support as the outcome; (3) terms that exclude letters to the editors, notes and editorials; and (4) terms to exclude items published prior to 1 January 2004.

We will complement our search by screening reference lists of reviews related to the topic and of included studies and their citations through Scopus, following Bramer.[20] We will update our search to add the most recent reports just before submitting our final review report for publication.

Study records

Data management

All records identified by the search strategy will be extracted into an Endnote Library, and we will de-duplicate using this software following the procedure outlined by Bramer.[21] If any duplicates remain those will be manually excluded. At this stage, duplicates will be identified based on overlapping author names and titles. The total number of detected duplicates will be noted in the final report.

Selection process

After removing duplicates, titles and abstracts of records identified during the literature search will be screened independently for inclusion by two reviewers. After initial selection based on screening of titles and abstracts, full-text articles will be screened for eligibility according to the inclusion and exclusion criteria by two reviewers, and discrepancies will be resolved after discussion with a third reviewer. Remaining duplicates based on populations, sample size and reported outcomes will be identified based on full text. The reviewers will not be blinded to information about the articles (e.g. authors' names and affiliations) at any stage.

Data collection process

Two reviewers will independently extract relevant data from all included studies according to a customised data extraction form developed a *priori* that was piloted using four eligible studies. Upon completion the reviewers will compare their results and any discrepancies will again be resolved after discussion with a third reviewer. If any relevant data are missing the corresponding authors will be contacted.

For peer review only

Data items

Customised data extraction forms will be used to extract relevant information from the eligible studies, which will include the following items:

(1) First author's name and affiliation
(2) Publication year
(3) Type of publication
(4) Access information (DOI or URL)
(5) Study design
(6) Location of the study (e.g. country, region)
(7) Description of the policy (e.g. places covered, whether or not the intervention is implemented, national or regional/local implementation)
(8) For studies assessing support for policies already implemented:
    a) date of implementation,
    b) level of implementation (e.g. government, municipality),
    c) level of enforcement (e.g. voluntary, warnings, fines)
(9) Observational period
(10) Selection of participants (e.g. eligibility criteria, sampling methods)
(11) Number of participants
(12) Data source (e.g. national survey, study recruited participants)
(13) Method of data collection
(14) Definition of public support
(15) Statistical analyses (if applicable)
(16) Number and percentages of missing values and non-response (if applicable)
(17) Techniques for handling missing values and non-response
(18) Characteristics of the study population (e.g. age, gender, smoking status)
(19) Levels of public support (estimate, 95% confidence intervals)
(20) Determinants of public support (see section *data synthesis* for more detail)
    a) Within-study determinants
    b) Between-study determinants
    c) Context-specific determinants
(21) Any conflict of interest reported by the authors
(22) Funding source(s)

Data will be complemented with the World Bank Country Classification by income, based on Gross National Income (GNI) per capita.[22] Furthermore, we will seek information regarding whether at the time of the study traditional smoke-free regulation was already implemented in enclosed public areas and workspaces according to the WHO.[17]

Outcomes and prioritisation

Data will be extracted for each estimate of public support by the spaces that they cover (e.g. playgrounds, private cars, multi-unit housing). If weighted and unweighted estimates are presented, we will extract estimates that are weighted to most adequately reflect the general population. If multiple estimates are presented that relate to public support, we will extract the estimate that covers the most general spaces. For example, we will prioritise "it should be illegal to smoke in all playgrounds" above "it

For peer review only

should be illegal to smoke in this specific playground". If public support is asked in general and specifically related to children, we will extract both estimates. For example, we will extract "it should be illegal to smoke in private cars" and "it should be illegal to smoke in private cars when minors are present".

Risk of bias assessment

We will assess risk of bias for each study using the Mixed Methods Appraisal Tool (MMAT) for descriptive studies. The following five elements will be assessed: relevance of the sampling strategy, representativeness of the target population, appropriateness of the outcome measurements, risk of non-response bias, and appropriateness of the statistical techniques. Each of the elements will be categorised by using the answer categories *yes*, *no* or *can't tell*, following MMAT criteria. Results of the risk-of-bias analysis will be presented in tables.[23]

Data synthesis

Obtaining comparable data is essential to facilitate meta-analysis, thus homogenisation of the outcome data is needed. Public support will be analysed as proportional data, i.e. proportion of the population supporting a particular smoke-free policy. The outcome estimates will be reversed if studies report on the proportion not in favour of the smoke-free policies. Often Likert-scale type questions are used to assess support, if studies report percentages per answer option instead of total support, the answer categories above neutral (i.e. indicating a positive response) will be combined.

To allow meta-analyses, standard errors (SE) are needed. If SE are not presented they will be calculated using the following formula;[24]

$$fraction = \frac{nr\ of\ people\ supporting\ policy}{sample\ size}$$

$$SE = \sqrt{\frac{fraction(1 - fraction)}{sample\ size}}$$

Two reviewers will independently assess whether measures of public support and smoke-free policies under investigation are sufficiently comparable across the selected studies to allow meta-analysis. If needed, they will convert the units of measurement in a way that is consistent across studies. In case of disagreement, a third reviewer will decide which measures to use.

Prior to undertaking meta-analyses, we will subdivide policies by spaces that they cover according to the following division: (1) indoor private spaces (e.g. cars), (2) indoor semi-private spaces (e.g. multi-unit housing), (3) outdoor (semi-)private spaces (e.g. courtyard), (4) non-hospitality outdoor public spaces (e.g. parks, streets, beaches, hospital grounds, playgrounds), and (5) hospitality outdoor public spaces (e.g. restaurant terraces). Separate meta-analyses will be conducted to assess public support for smoke-free policies according to these categories. If multiple estimates of public support are presented that cover similar spaces according to our categorisation, we will calculate the average public support across these spaces for use in meta-analyses. Thus, if studies present separate estimates of public support for

For peer review only

playgrounds, parks and beaches (all belonging to the category 'non-hospitality outdoor public spaces'), the average of the three will be used. In case of overlapping study populations, we will include the study or effect estimation that: (1) uses national surveys and therefore is likely to be representative of the general population, (2) has the lowest risk of bias, or (3) incorporates the largest sample size, following this hierarchy. We aim to summarise the most up-to-date status of public support for novel smoke-free policies; thus, we will include the most recent estimation if studies presented multiple estimates over time. In secondary analyses we will evaluate whether public support changed following the actual introduction of the smoke-free policy under study, if the data allows.

One of the assumptions in meta-analysis is that effect sizes are independent, i.e. the effect size of one study does not imply the direction or magnitude of the effect size in another study.[25] Multiple estimates of public support in a specific country may violate the independence assumption, therefore we will conduct a three-level meta-analysis.[26] A three-level meta-analysis is an extended version of a random-effects meta-analysis and includes sampling variation at the first level, within-country heterogeneity at the second level and between-country heterogeneity at the third level. The analytic model is as follows:

$$(1)\quad \hat{\theta}_{ij} = \beta_0 + \zeta_{(2)ij} + \zeta_{(3)j} + \epsilon_{ij}.$$

Where $\hat{\theta}_{ij}$ is the estimation of the true effect size for public support, β_0 the average population effect, $\zeta_{(2)ij}$ is the within-country variance, $\zeta_{(3)j}$ is the between-country variance, and ϵ_{ij} the sampling variance.[26] In each model, heterogeneity will be quantified by the I^2 statistic per level.

A second analysis will be performed on all studies presenting the change in public support following implementation of the actual policy under study. If relevant analyses will be performed twice, once including estimates for public support in general and once for public support specifically related to children, e.g. public support for smoking bans in cars and public support for smoking bans in cars if children are present (see section *outcomes and prioritisation)*.

The secondary aim is to identify and quantify determinants that are associated with public support. The determinants of public support will be evaluated at three levels:

1. Quantify within-study determinants of public support

Public support may differ between population subgroups. Therefore, we will conduct subgroup analyses according to:

- Gender (men vs. women)
- Smoking status (current smokers vs. former smokers vs. non-smokers, and/or current smokers vs. non-smokers (including former smokers), depending on data availability)
- Parental status (yes vs. no, depending on data availability)
- Age group (younger vs. older, categorisation depending on data availability)

Public support will be pooled per subgroup for each of the five space categories using three-level meta-analysis.

2. Quantify between-study determinants of public support

Various study-specific elements may influence public support. Meta-regression analysis will be performed to assess between-study determinants of public support per space category. In these

For peer review only

analyses support in favour of smoke-free policies is used as dependent variable and the following variables per study are used as independent variables:

- Calendar year in which the survey was conducted (continuous);
- Whether public support was assessed as yes-no or on a Likert-scale (binary);
- Income level of the country, according to the World Bank classification (binary: high- versus low- and middle-income countries); and
- Whether or not traditional-smoke-free legislation covering enclosed public places and workplaces was in place (categorical: none, partial, or comprehensive according to the WHO classification[17]).

3. Identify context-specific determinants of public support

Context-specific determinants of public support will be identified using thematic synthesis. We will follow the method outlined by Thomas and Harden[27] consisting of three steps: (1) coding text, (2) developing descriptive themes and (3) generating analytic themes. The full-text of each study will be extracted and uploaded into NVivo V.12 (NVivo Qualitative data analysis software V.10: QSR International Pty Ltd, 2012). As studies may provide information outside the scope of this review, coding will be limited to sentences describing details that relate to determinants of public support for smoke-free policies. In order to ensure a consistent coding methodology, three eligible articles will be coded independently by two reviewers and then compared until consensus on the themes has been reached. The remaining articles will be coded independently by two reviewers. After every five articles coding will be compared to ensure consistency. *A priori* four core domains have been identified: (1) beliefs and scientific evidence about effectiveness, (2) social norms, (3) communication and implementation strategies, and (4) collaboration between stakeholders.

Sensitivity analysis

Study findings may vary according to the risk-of-bias level of the individual studies. As a sensitivity analysis, we will exclude studies that scored *no* or *can't tell* on at least one domain following the MMAT criteria. This criterion is based on MMAT evaluations in previous literature.[28]

Ethics and dissemination

No primary data collection will be undertaken; therefore, no formal ethical assessment and informed consent are required. Findings will be summarised in a single manuscript and will be disseminated through scientific literature.

Timeline

Start date: April 15th 2020

Finishing date: 31 December 2020

Reporting date:

Authors contributions

For peer review only

JVB conceptualised the study and secured funding. All authors contributed to the design of the protocol. NWB and FJMM wrote the first draft and revised subsequent drafts. JVB supervised the writing. All authors contributed to the writing and have read and approved the final manuscript.

Funding statement

This work was funded by a joint collaboration between Lung Foundation Netherlands, Dutch Cancer Society, Dutch Heart Foundation, Dutch Diabetes Research Foundation and the Netherlands Thrombosis Foundation. AS is supported by Health Data Research UK.

Competing interests

The authors declare that they have no competing interests.

References

1. Stanaway JD, Afshin A, Gakidou E, et al. Global, regional, and national comparative risk assessment of 84 behavioural, environmental and occupational, and metabolic risks or clusters of risks for 195 countries and territories, 1990–2017: a systematic analysis for the Global Burden of Disease Study 2017. *Lancet* 2018;**392**(10159):1923-94.
2. Hoffman SJ, Tan C. Overview of systematic reviews on the health-related effects of government tobacco control policies. *BMC Public Health* 2015;**15**:744.
3. Faber T, Kumar A, Mackenbach JP, et al. Effect of tobacco control policies on perinatal and child health: a systematic review and meta-analysis. *The Lancet Public Health* 2017;**2**(9):e420-e37.
4. Frazer K, Callinan JE, McHugh J, et al. Legislative smoking bans for reducing harms from secondhand smoke exposure, smoking prevalence and tobacco consumption. *Cochrane Database Syst Rev* 2016;**2**:CD005992.
5. World Health Organization. WHO Report on the Global Tobacco Epidemic, 2009: implemeting smoke-free environments France: Geneva: World Health Organization, 2009.
6. Allwright S. Republic of Ireland's indoor workplace smoking ban: British Journal of General Practice, 2004.
7. Thomson G, Wilson N. Public attitudes to laws for smoke-free private vehicles: a brief review. *Tobacco control* 2009;**18**(4):256-61.
8. Johns M, Coady MH, Chan CA, et al. Evaluating New York City's smoke-free parks and beaches law: A critical multiplist approach to assessing behavioral impact. *American journal of community psychology* 2013;**51**(1-2):254-63.
9. Levy DE, Adams IF, Adamkiewicz G. Delivering on the Promise of Smoke-Free Public Housing. *American journal of public health* 2017;**107**(3):380-83.
10. International Agency for Research on Cancer World Health Organization. Evaluating the effectiveness of smoke-free policies. Lyon: IARC Press, International Agency for Research on Cancer, 2009.
11. Thomson G, Wilson N, Collins D, et al. Attitudes to smoke-free outdoor regulations in the USA and Canada: a review of 89 surveys. *Tobacco Control* 2016;**25**(5):506-16.
12. Fong GT, Hyland A, Borland R, et al. Reductions in tobacco smoke pollution and increases in support for smoke-free public places following the implementation of comprehensive smoke-free workplace legislation in the Republic of Ireland: findings from the ITC Ireland/UK Survey. *Tob Control* 2006;**15**(suppl 3):iii51.

For peer review only

13. Fabian LE, Bernat DH, Lenk KM, et al. Smoke-free laws in bars and restaurants: does support among teens and young adults change after a statewide smoke-free law? *Public Health Rep* 2011;**126**(5):669-76.
14. Thomson G, Wilson N. One year of smokefree bars and restaurants in New Zealand: impacts and responses. *BMC Public Health* 2006;**6**(1):64.
15. Kuijpers TG, Willemsen MC, Kunst AE. Public support for tobacco control policies: The role of the protection of children against tobacco. *Health Policy* 2018;**122**(8):929-35.
16. Rozema AD, Mathijssen JJP, Jansen MWJ, et al. Schools as smoke-free zones? Barriers and facilitators to the adoption of outdoor school ground smoking bans at secondary schools. *Tob Induc Dis* 2016;**14**(1):10.
17. World Health Organization. Protection from exposure to second-hand tobacco smoke: policy recommendations, 2007.
18. Bartlett JE, Kotrlik JW, Higgins CCH. Organizational research: Determining appropriate sample size in survey research appropriate sample size in survey research. *Inf Technol J* 2001;**19**(1):8.
19. Thomson G, Wilson N, Collins D, et al. Attitudes to smoke-free outdoor regulations in the USA and Canada: a review of 89 surveys. *Tob Control* 2016;**25**(5):506-16.
20. Bramer WM. Reference checking for systematic reviews using Endnote. *J Med Libr Assoc* 2018;**106**(4):542-46.
21. Bramer WM, Giustini D, de Jonge GB, et al. De-duplication of database search results for systematic reviews in EndNote. *Journal of the Medical Library Association: JMLA* 2016;**104**(3):240.
22. World Bank. Country Classification In: Group TWB, ed., 2019.
23. Hong QN, Pluye P, Fàbregues S, et al. Mixed methods appraisal tool (MMAT), version 2018. *IC Canadian Intellectual Property Office, Industry Canada* 2018.
24. Nyaga VN, Arbyn M, Aerts M. Metaprop: a Stata command to perform meta-analysis of binomial data. *Archives of Public Health* 2014;**72**(1):39.
25. Van den Noortgate W, Lopez-Lopez JA, Marin-Martinez F, et al. Three-level meta-analysis of dependent effect sizes. *Behav Res Methods* 2013;**45**(2):576-94.
26. Assink M, Wibbelink CJM. Fitting three-level meta-analytic models in R: A step-by-step tutorial. *The Quantitative Methods for Psychology* 2016;**12**(3):154-74.
27. Thomas J, Harden A. Methods for the thematic synthesis of qualitative research in systematic reviews. *BMC Medical Research Methodology* 2008;**8**(1):45.
28. Eykelenboom M, van Stralen MM, Olthof MR, et al. Political and public acceptability of a sugar-sweetened beverages tax: a mixed-method systematic review and meta-analysis. *International Journal of Behavioral Nutrition and Physical Activity* 2019;**16**(1):78.

For peer review only

Appendix I

embase.com (1974-)

('smoking regulation'/exp OR (smokefree OR ((smoking OR smoke OR tobacco) NEAR/3 (regulation* OR government* OR law OR laws OR policy OR policies OR ban OR bans OR banned OR free OR restrict* OR act OR acts))):ab,ti) AND ('public opinion'/exp OR 'public attitude'/de OR (opinion* OR support* OR views OR (public NEAR/3 view) OR attitude* OR feeling* OR acceptance* OR accepts OR perception* OR misperception*):ab,ti) NOT ([Letter]/lim OR [Note]/lim OR [Editorial]/lim)

Medline Ovid (1946-)

(Smoke-Free Policy/ OR (smokefree OR ((smoking OR smoke OR tobacco) ADJ3 (regulation* OR government* OR law OR laws OR policy OR policies OR ban OR bans OR banned OR free OR restrict* OR act OR acts))).ab,ti.) AND (Public Opinion/ OR Attitude/ OR (opinion* OR support* OR views OR (public ADJ3 view) OR attitude* OR feeling* OR acceptance* OR accepts OR perception* OR misperception*).ab,ti.) NOT (news OR book* OR chapter* OR dissertation abstract*).pt.

Web of science Core Collection (1975-)

TS=(((smokefree OR ((smoking OR smoke OR tobacco) NEAR/2 (regulation* OR government* OR law OR laws OR policy OR policies OR ban OR bans OR banned OR free OR restrict* OR act OR acts)))) AND ((opinion* OR support* OR views OR (public NEAR/2 view) OR attitude* OR feeling* OR acceptance* OR accepts OR perception* OR misperception*)))

PsycINFO Ovid (1806-)

((smokefree OR ((smoking OR smoke OR tobacco) ADJ3 (regulation* OR government* OR law OR laws OR policy OR policies OR ban OR bans OR banned OR free OR restrict* OR act OR acts))).ab,ti.) AND (Public Opinion/ OR Attitudes/ OR (opinion* OR support* OR views OR (public ADJ3 view) OR attitude* OR feeling* OR acceptance* OR accepts OR perception* OR misperception*).ab,ti.) NOT (news OR book* OR chapter* OR dissertation abstract*).pt.

LILACS

(smokefree OR smoking OR smoke) AND (opinions OR views OR attitudes)

Scientific Electronic Library Online [SciELO])

(smokefree OR smoking OR smoke) AND (opinions OR views OR attitudes)

WHO Global Health Library

(smokefree OR smoking OR smoke) AND (opinions OR views OR attitudes)

For peer review only

PRISMA-P (Preferred Reporting Items for Systematic review and Meta-Analysis Protocols) 2015 checklist: recommended items to address in a systematic review protocol*

Section and topic	Item No	Checklist item	Page
**ADMINISTRATIVE INFORMATION**			
Title:			
Identification	1a	Identify the report as a protocol of a systematic review	1
Update	1b	If the protocol is for an update of a previous systematic review, identify as such	N.A.
Registration	2	If registered, provide the name of the registry (such as PROSPERO) and registration number	2
Authors:			
Contact	3a	Provide name, institutional affiliation, e-mail address of all protocol authors; provide physical mailing address of corresponding author	1
Contributions	3b	Describe contributions of protocol authors and identify the guarantor of the review	10
Amendments	4	If the protocol represents an amendment of a previously completed or published protocol, identify as such and list changes; otherwise, state plan for documenting important protocol amendments	N.A.
Support:			
Sources	5a	Indicate sources of financial or other support for the review	10
Sponsor	5b	Provide name for the review funder and/or sponsor	10
Role of sponsor or funder	5c	Describe roles of funder(s), sponsor(s), and/or institution(s), if any, in developing the protocol	10
**INTRODUCTION**			
Rationale	6	Describe the rationale for the review in the context of what is already known	3
Objectives	7	Provide an explicit statement of the question(s) the review will address with reference to participants, interventions, comparators, and outcomes (PICO)	4
**METHODS**			
Eligibility criteria	8	Specify the study characteristics (such as PICO, study design, setting, time frame) and report characteristics (such as years considered, language, publication status) to be used as criteria for eligibility for the review	4
Information sources	9	Describe all intended information sources (such as electronic databases, contact with study authors, trial registers or other grey literature sources) with planned dates of coverage	5
Search strategy	10	Present draft of search strategy to be used for at least one electronic database, including planned limits, such that it could be repeated	Appendix I

Study records:			
Data management	11a	Describe the mechanism(s) that will be used to manage records and data throughout the review	5
Selection process	11b	State the process that will be used for selecting studies (such as two independent reviewers) through each phase of the review (that is, screening, eligibility and inclusion in meta-analysis)	5
Data collection process	11c	Describe planned method of extracting data from reports (such as piloting forms, done independently, in duplicate), any processes for obtaining and confirming data from investigators	5
Data items	12	List and define all variables for which data will be sought (such as PICO items, funding sources), any pre-planned data assumptions and simplifications	6
Outcomes and prioritization	13	List and define all outcomes for which data will be sought, including prioritization of main and additional outcomes, with rationale	6
Risk of bias in individual studies	14	Describe anticipated methods for assessing risk of bias of individual studies, including whether this will be done at the outcome or study level, or both; state how this information will be used in data synthesis	7
Data synthesis	15a	Describe criteria under which study data will be quantitatively synthesised	7
	15b	If data are appropriate for quantitative synthesis, describe planned summary measures, methods of handling data and methods of combining data from studies, including any planned exploration of consistency (such as I^2, Kendall's τ)	7
	15c	Describe any proposed additional analyses (such as sensitivity or subgroup analyses, meta-regression)	8
	15d	If quantitative synthesis is not appropriate, describe the type of summary planned	N.A.
Meta-bias(es)	16	Specify any planned assessment of meta-bias(es) (such as publication bias across studies, selective reporting within studies)	9
Confidence in cumulative evidence	17	Describe how the strength of the body of evidence will be assessed (such as GRADE)	-

*** It is strongly recommended that this checklist be read in conjunction with the PRISMA-P Explanation and Elaboration (cite when available) for important clarification on the items. Amendments to a review protocol should be tracked and dated. The copyright for PRISMA-P (including checklist) is held by the PRISMA-P Group and is distributed under a Creative Commons Attribution Licence 4.0.**

From: Shamseer L, Moher D, Clarke M, Ghersi D, Liberati A, Petticrew M, Shekelle P, Stewart L, PRISMA-P Group. Preferred reporting items for systematic review and meta-analysis protocols (PRISMA-P) 2015: elaboration and explanation. BMJ. 2015 Jan 2;349(jan02 1):g7647.

For peer review only

BMJ Open

Assessing public support for extending smoke-free policies beyond enclosed public places and workplaces: protocol for a systematic review and meta-analysis

Journal:	*BMJ Open*
Manuscript ID	bmjopen-2020-040167.R1
Article Type:	Protocol
Date Submitted by the Author:	20-Oct-2020
Complete List of Authors:	Boderie, Nienke; Erasmus MC, University Medical Centre Rotterdam, Department of Public Health<br>Mölenberg, Famke; Erasmus MC, University Medical Centre Rotterdam, Department of Public Health<br>Sheikh, Aziz; University of Edinburgh, Centre of Medical Informatics, Usher Institute ; Brigham and Women's Hospital/Harvard Medical School, Division of General Internal Medicine and Primary Care<br>Bramer, Wichor; Erasmus MC, University Medical Centre Rotterdam, Medical Library<br>Burdorf, Alex; Erasmus MC, University Medical Centre Rotterdam, Department of Public Health<br>Van Lenthe, Frank; Erasmus MC, University Medical Centre Rotterdam, Department of Public Health<br>Been, Jasper; Erasmus MC, University Medical Centre Rotterdam, Department of Public Health; Erasmus MC - Sophia Children's Hospital, University Medical Centre Rotterdam, Department of Paediatrics
**Primary Subject Heading**:	Public health
Secondary Subject Heading:	Health policy, Public health
Keywords:	PUBLIC HEALTH, PREVENTIVE MEDICINE, Health policy < HEALTH SERVICES ADMINISTRATION & MANAGEMENT

SCHOLARONE™
Manuscripts

I, the Submitting Author has the right to grant and does grant on behalf of all authors of the Work (as defined in the below author licence), an exclusive licence and/or a non-exclusive licence for contributions from authors who are: i) UK Crown employees; ii) where BMJ has agreed a CC-BY licence shall apply, and/or iii) in accordance with the terms applicable for US Federal Government officers or employees acting as part of their official duties; on a worldwide, perpetual, irrevocable, royalty-free basis to BMJ Publishing Group Ltd ("BMJ") its licensees and where the relevant Journal is co-owned by BMJ to the co-owners of the Journal, to publish the Work in this journal and any other BMJ products and to exploit all rights, as set out in our licence.

The Submitting Author accepts and understands that any supply made under these terms is made by BMJ to the Submitting Author unless you are acting as an employee on behalf of your employer or a postgraduate student of an affiliated institution which is paying any applicable article publishing charge ("APC") for Open Access articles. Where the Submitting Author wishes to make the Work available on an Open Access basis (and intends to pay the relevant APC), the terms of reuse of such Open Access shall be governed by a Creative Commons licence – details of these licences and which Creative Commons licence will apply to this Work are set out in our licence referred to above.

Other than as permitted in any relevant BMJ Author's Self Archiving Policies, I confirm this Work has not been accepted for publication elsewhere, is not being considered for publication elsewhere and does not duplicate material already published. I confirm all authors consent to publication of this Work and authorise the granting of this licence.

For peer review only

Assessing public support for extending smoke-free policies beyond enclosed public places and workplaces: protocol for a systematic review and meta-analysis

Registration number …

Nienke W. Boderie[1], Famke J.M. Mölenberg[1], Aziz Sheikh[2,3], Wichor M. Bramer[4], Alex Burdorf[1], Frank J. van Lenthe[1], Jasper V. Been[1,2,5]

[1] Department of Public Health, Erasmus MC, University Medical Centre Rotterdam, Rotterdam, Netherlands
[2] Centre of Medical Informatics, Usher Institute, The University of Edinburgh, Edinburgh, UK
[3] Division of General Internal Medicine and Primary Care, Brigham and Women's Hospital/Harvard Medical School, Boston, MA, USA
[4] Medical Library, Erasmus MC, University Medical Centre Rotterdam, Rotterdam, Netherlands
[5] Department of Paediatrics, division of Neonatology, Erasmus MC - Sophia Children's Hospital, University Medical Centre Rotterdam, Rotterdam, Netherlands

Corresponding author:
Jasper V. Been
Erasmus University Medical Centre, PO Box 2040, 3000 CA Rotterdam
j.been@erasmusmc.nl
+31 10 70 36077

Word count: 3417

For peer review only - http://bmjopen.bmj.com/site/about/guidelines.xhtml

KEYWORDS: Smoke-Free Policy, Tobacco Smoke Pollution, Attitude

ABSTRACT: (296 words)

Introduction: Smoke-free enclosed public environments are effective in reducing exposure to second-hand smoke and yield major public health benefits. Building on this, many countries are now implementing smoke-free policies regulating smoking beyond enclosed public places and workplaces. In order to successfully implement such 'novel smoke-free policies' public support is essential. We aim to provide the first comprehensive systematic review and meta-analysis assessing levels and determinants of public support for novel smoke-free policies.

Methods and analysis: The primary objective of this review is to summarise the level of public support for novel smoke-free policies. Eight online databases (from 1 January 2004, no language restrictions) will be searched by two independent researchers. Studies are eligible if assessing support for novel smoke-free policies in the general population (age ≥16 years) and have a sample size of n≥400. Studies funded by the tobacco industry or evaluating support among groups with vested interest are excluded. The primary outcome is proportion of public support for smoke-free policies, subdivided according to the spaces covered: (1) indoor private spaces (e.g. cars), (2) indoor semi-private spaces (e.g. multi-unit housing), (3) outdoor (semi-)private spaces (e.g. courtyards), (4) non-hospitality outdoor public spaces (e.g. parks, hospital grounds, playgrounds), and (5) hospitality outdoor public spaces (e.g. restaurant terraces).The secondary objective is to identify determinants associated with public support on three levels: (1) within-study determinants (e.g. smoking status), (2) between-study determinants (e.g. survey year), and (3) context-specific determinants (e.g. social norms). Risk of bias will be assessed using the Mixed Methods Appraisal Tool (MMAT) and a sensitivity analysis will be performed excluding studies at high-risk-of-bias.

Ethics and dissemination: No formal ethical approval is required. Findings will be disseminated to academics, policy-makers and the general public.

The protocol is registered with PROSPERO registration number: x

STRENGHTS AND LIMITATIONS

- This systematic review is unique in providing a structured overview of levels of public support for 'novel smoke-free policies' (i.e. smoke free policies that go beyond regulating smoking in enclosed public places and workplaces)
- Within- and between-study determinants associated with public support will be assessed, and thematic synthesis will be used to identify context-specific determinants.
- The protocol presented has been designed in line with the Preferred Reporting Items for Systematic Reviews and Meta-Analysis for Protocols (PRISMA-P)
- The generalizability and value of this systematic review depends on the availability and quality of the data.

For peer review only - http://bmjopen.bmj.com/site/about/guidelines.xhtml

For peer review only

INTRODUCTION

Second-hand smoke (SHS) exposure is related to 1.2 million deaths per year.[1] Smoke-free environments have proven to be effective in reducing exposure to SHS and have major public health benefits.[2] Previous systematic reviews reported consistent evidence for improved cardiovascular health and reduced smoking-related mortality, as well as reductions in preterm birth, severe asthma exacerbations and respiratory tract infections in children, following implementation of smoke-free legislation in indoor public places and workplaces.[3-5] It has been shown that outdoor areas contribute significantly to SHS exposure, therefore the implementation of smoking-free policies in open spaces have the potential to reduce the associated burden of disease.[6,7]

In 2004, Ireland was the first country in the world to implement comprehensive smoke-free legislation covering enclosed workplaces and public places, and many more countries followed its example.[8] An increasing number of jurisdictions is now implementing, or considering implementing, additional smoke-free policies that go beyond regulating smoking in enclosed public places and target private and outdoor spaces, henceforth referred to as 'novel smoke-free policies'. Novel smoke-free policies are implemented in an attempt to further improve population health via reducing SHS exposure. For example, several countries have implemented laws requiring private cars carrying children be smoke-free,[9-11] smoke free hospital campuses have been implemented country wide in Spain and Ireland,[12,13] the city of New York banned smoking in all public parks, pedestrian plazas and at all beaches,[14] and the US Department of Public Housing and Urban Development requires all public housing units to be smoke-free, both within resident units and in public areas.[15] Public support is essential in democracies in order for policy-makers to consider implementing such novel smoke-free policies and to increase the likelihood of successful implementation,[16] and accordingly the World Health Organisation (WHO) stated that "Involving civil society is central to achieving effective legislation".[5]

However, pubic support may vary over time, as well as by population subgroups. For example, women and non-smokers tend to be more in favour of smoke-free legislation than men and current smokers.[17] Several studies showed that public support for smoke-free policies increased after successful implementation and particularly so among smokers.[18-20] Furthermore, public support for smoke-free policies was higher when policies covered spaces that were frequently visited by those more vulnerable to the adverse health effects of SHS.[21] For example, in the US and Canada public support for smoke-free playgrounds (89-91%) was substantially higher than for smoke-free outdoor workplaces (12-46%) and sidewalks (31-49%).[17] Context-specific determinants may also contribute to differences in public support across settings. Aspects that enhanced successful adoption of smoke-free zones at outdoor school grounds at secondary schools included communication about the policy, collaboration between and within stakeholders, social norms, and evidence about the effectiveness of smoke-free zones.[22]

A structured overview of the levels and determinants of public support for smoke-free policies beyond enclosed public places and workplaces across various settings is currently lacking. Having these insights may guide policy makers with the implementation of policies that receive the highest levels of support, and may help in defining additional strategies that are needed to increase public support in the population. To address this gap in the literature our primary objective is to summarise the level of public support across the globe for novel smoke-free policies and to evaluate if public support changed following implementation of the novel smoke-free policies across various settings. To do so a systematic review and meta-analysis will be conducted. The secondary objective is to identify determinants associated with public support at the following three levels: 1) within-study determinants (e.g. age,

For peer review only - http://bmjopen.bmj.com/site/about/guidelines.xhtml

smoking status, parental status), 2) between-study determinants (e.g. income level of the country, whether smoke-free legislation in enclosed public places and workplaces was already in place), and 3) context-specific determinants (e.g. setting, framing, enforcement of smoke-free policies).

METHODS AND ANALYSIS

We used the Preferred Reporting Items for Systematic Reviews and Meta-Analysis Protocols (PRISMA-P) guidelines to facilitate development of this protocol, see appendix I. The protocol has been registered in PROSPERO as …

In this review, we will use the term 'traditional smoke-free legislation' to refer to smoke-free legislation covering enclosed public places and workplaces (i.e. compliant with Article 8(2) of the Framework Convention on Tobacco Control; FCTC) and the term 'novel smoke-free policies' to refer to policies and legislation regulating smoking in any other places, such as (semi)private places and (partially) outdoor spaces, whether public or (semi)private.[23] Policies are used in the broadest sense and are not necessarily enacted via formal legislation as this will allow us to evaluate less formal local smoke-free initiatives (e.g. self-regulation by the hospitality sector or local hospitals) as well as formal legislation.

Eligibility criteria

We will include articles published in scientific journals as well as 'grey literature' evaluating public support for novel smoke-free policies covering (semi)private places and (partially) outdoor spaces, whether public or (semi)private. Grey literature includes policy documents and reports that are published non-commercially and/or are not indexed by major scientific literature databases. Cohort studies and (repeated) cross-sectional studies will be included, quantitative studies will be excluded and no language restrictions are applied. We will seek translation for reports in foreign languages to assess eligibility. Studies for which only an abstract is available, will not be included since risk of bias for these studies cannot be adequately assessed.

Eligibility of the studies will be assessed using the following criteria:

(1) Studies will be eligible if support for one or more novel smoke-free policies is evaluated. We will include studies assessing support for novel smoke-free policies that are already in place as well as those assessing support for upcoming or theoretical implementation of such policies. Policies at any level are eligible, such as city-level, state-level, country level etc.. Studies will be excluded if solely evaluating traditional smoke-free legislation.[23]

(2) Studies will be eligible if they assessed public support for smoke-free policies in the population aged 16 years or above who represent the majority of a population primarily affected by the policy (e.g. support for a country wide measure is evaluated in a representative sample of the country, while support for a policy at a local campus is assessed among students and staff of that specific campus), or in any of the predefined population subgroups (see *within-study determinants of public support* below). We set this age criterion to include the part of the population that is entitled to vote in most democracies, and as such may be regarded to be of particular interest to politicians and policymakers. Any study reporting (sub)populations in which at least 50% fits this age criterion will also be included.

(3) Our primary objective is to summarise the level of public support for novel smoke free policies in the general population, therefore we will only include studies of which we can be confident

For peer review only - http://bmjopen.bmj.com/site/about/guidelines.xhtml

that the reported support in the study sample reflects the levels of support that would be found if the entire population was surveyed. This is operationalized by only including studies can ensure a 5% margin of error. Following sample size calculations for surveys, [24,25] a minimum sample size of 400 is required. Similar criteria was used by an earlier reviews assessing public support for outdoor smoke-free areas.[17]

(4) Studies will be included when published from 1 Jan 2004 onwards. This pragmatic cut-off was chosen as the first national traditional smoke-free law covering indoor public places and workplaces was introduced in Ireland in 2004. Hence, assessments of public support for novel smoke-free policies are unlikely to have preceded 2004, and are unlikely to be relevant for current everyday practice if they have.

(5) Studies will be excluded if solely evaluating support among specific subgroups not representing the majority of the population primarily affected by the policy, policy makers or groups with clearly vested interest, e.g. opinion of tobacco industry groups.

(6) Studies will be excluded when funded or supported by the tobacco industry, as the tobacco industry is known to "produce, sponsor and disseminate misleading research and information, lacking sound scientific methods". [26]

(7) Studies will be excluded if solely evaluating support for tobacco related subgroups, e.g. e-cigarettes or heatless tobacco products.

Information sources

The following electronic databases will be searched for eligible studies: (1) embase.com, (2) Medline ALL Ovid, (3) Web of Science Core Collection, (4) WHO Library Database (WHOLIS), (5) Latin American and Caribbean Health Sciences Literature (LILACS), (6) Scientific Online Library Online (SciELO), (7) PsychINFO and (8) Google Scholar.

Search strategy

The specific search strategies per database have been created in close collaboration with a bibliographic expert of the Erasmus MC with expertise in systematic reviewing (WMB; see Appendix 1). Search terms include three parts: (1) terms to identify smoke-free policies; (2) terms to identify measures of public support as the outcome; (3) terms that exclude letters to the editors, notes and editorials; and (4) terms to exclude items published prior to 1 January 2004.

We will complement our search by screening reference lists of reviews related to the topic and of included studies and their citations through Scopus, following Bramer.[27] We will update our search to add the most recent reports just before submitting our final review report for publication.

Study records

Data management

All records identified by the search strategy will be extracted into an Endnote Library, and we will de-duplicate using this software following the procedure outlined by Bramer. [28] If any duplicates remain those will be manually excluded. At this stage, duplicates will be identified based on overlapping author names and titles. The total number of detected duplicates will be noted in the final report.

For peer review only - http://bmjopen.bmj.com/site/about/guidelines.xhtml

Selection process

After removing duplicates, titles and abstracts of records identified during the literature search will be screened independently for inclusion by two reviewers. After initial selection based on screening of titles and abstracts, full-text articles will be screened for eligibility according to the inclusion and exclusion criteria by two reviewers, and discrepancies will be resolved after discussion with a third reviewer. Remaining duplicates based on populations, sample size and reported outcomes will be identified based on full text. The reviewers will not be blinded to information about the articles (e.g. authors' names and affiliations) at any stage.

Data collection process

Two reviewers will independently extract relevant data from all included studies according to a customised data extraction form developed a *priori* that was piloted using four eligible studies. Upon completion the reviewers will compare their results and any discrepancies will again be resolved after discussion with a third reviewer. If any relevant data are missing the corresponding authors will be contacted.

Data items

Customised data extraction forms will be used to extract relevant information from the eligible studies, which will include the following items:

(1) First author's name and affiliation
(2) Publication year
(3) Type of publication
(4) Access information (DOI or URL)
(5) Study design
(6) Location of the study (e.g. country, region)
(7) Description of the policy (e.g. places covered, whether or not the intervention is implemented, national or regional/local implementation)
(8) For studies assessing support for policies already implemented:
   a) date of implementation,
   b) level of implementation (e.g. government, municipality),
   c) level of enforcement (e.g. voluntary, warnings, fines)
(9) Observational period
(10) Selection of participants (e.g. eligibility criteria, sampling methods)
(11) Number of participants
(12) Data source (e.g. national survey, study recruited participants)
(13) Method of data collection
(14) Definition of public support
(15) Statistical analyses (if applicable)
(16) Number and percentages of missing values and non-response (if applicable)
(17) Techniques for handling missing values and non-response
(18) Characteristics of the study population (e.g. age, gender, smoking status)
(19) Levels of public support (estimate, 95% confidence intervals)
(20) Determinants of public support (see section *data synthesis* for more detail)

For peer review only - http://bmjopen.bmj.com/site/about/guidelines.xhtml

For peer review only

a) Within-study determinants
b) Between-study determinants
c) Context-specific determinants

(21) Any conflict of interest reported by the authors
(22) Funding source(s)

Data will be complemented with the World Bank Country Classification by income, based on Gross National Income (GNI) per capita.[29] Furthermore, we will seek information regarding whether at the time of the study traditional smoke-free regulation was already implemented in enclosed public areas and workspaces according to the WHO.[23]

Outcomes and prioritisation

Data will be extracted for each estimate of public support by the spaces that they cover (e.g. playgrounds, private cars, multi-unit housing). If weighted and unweighted estimates are presented, we will extract estimates that are weighted to most adequately reflect the general population. If multiple estimates are presented that relate to public support, we will extract the estimate that covers the most general spaces. For example, we will prioritise “it should be illegal to smoke in all playgrounds” above “it should be illegal to smoke in this specific playground”. If public support is asked in general and specifically related to children, we will extract both estimates. For example, we will extract “it should be illegal to smoke in private cars” and “it should be illegal to smoke in private cars when minors are present”.

Risk of bias assessment

We will assess risk of bias for each study using the Mixed Methods Appraisal Tool (MMAT) for descriptive studies. The MMAT 2018 version was developed based on criteria from 18 existing critical appraisal tools and input from over 50 international experts. The following five elements will be assessed: relevance of the sampling strategy, representativeness of the target population, appropriateness of the outcome measurements, risk of non-response bias, and appropriateness of the statistical techniques. Each of the elements will be categorised by using the answer categories *yes*, *no* or *can’t tell*, following MMAT criteria. Results of the risk-of-bias analysis will be presented in tables.[30]

Data synthesis

Obtaining comparable data is essential to facilitate meta-analysis, thus homogenisation of the outcome data is needed. Public support will be analysed as proportional data, i.e. proportion of the population supporting a particular smoke-free policy. When results are fairly normal distributed the raw proportions will be analysed, if not logit transformations will be applied[31]The outcome estimates will be reversed if studies report on the proportion not in favour of the smoke-free policies. Often Likert-scale type questions are used to assess support, if studies report percentages per answer option instead of total support, the answer categories above neutral (i.e. indicating a positive response) will be combined.

To allow meta-analyses, standard errors (SE) are needed. If SE are not presented they will be calculated using the following formula;[32]

$$fraction = \frac{nr\ of\ people\ supporting\ policy}{sample\ size}$$

For peer review only - http://bmjopen.bmj.com/site/about/guidelines.xhtml

$$SE = \sqrt{\frac{fraction(1 - fraction)}{sample\ size}}$$

Two reviewers will independently assess whether measures of public support and smoke-free policies under investigation are sufficiently comparable across the selected studies to allow meta-analysis. If needed, they will convert the units of measurement in a way that is consistent across studies. In case of disagreement, a third reviewer will decide which measures to use.

Prior to undertaking meta-analyses, we will subdivide policies by spaces that they cover according to the following division: (1) indoor private spaces (e.g. cars), (2) indoor semi-private spaces (e.g. multi-unit housing), (3) outdoor (semi-)private spaces (e.g. courtyard, psychiatric hospital), (4) non-hospitality outdoor public spaces (e.g. parks, streets, beaches, hospital grounds, playgrounds), and (5) hospitality outdoor public spaces (e.g. restaurant terraces). Separate meta-analyses will be conducted to assess public support for smoke-free policies according to these categories. If multiple estimates of public support are presented that cover similar spaces according to our categorisation, we will calculate the average public support across these spaces for use in meta-analyses. Thus, if studies present separate estimates of public support for playgrounds, parks and beaches (all belonging to the category 'non-hospitality outdoor public spaces'), the average of the three will be used. In case of overlapping study samples, we will include the study or effect estimation that: (1) uses national surveys and therefore is likely to be representative of the general population, (2) has the lowest risk of bias, or (3) incorporates the largest sample size, following this hierarchy. We aim to summarise the most up-to-date status of public support for novel smoke-free policies; thus, we will include the most recent estimation if studies presented multiple estimates over time. In secondary analyses we will evaluate whether public support changed following the actual introduction of the smoke-free policy under study, if the data allows.

One of the assumptions in meta-analysis is that effect sizes are independent, i.e. the effect size of one study does not imply the direction or magnitude of the effect size in another study.[33] Multiple estimates of public support in a specific country may violate the independence assumption, therefore we will conduct a three-level meta-analysis.[34] A three-level meta-analysis is an extended version of a random-effects meta-analysis and includes sampling variation at the first level, within-country heterogeneity at the second level and between-country heterogeneity at the third level. The analytic model is as follows:

(1) $\hat{\theta}_{ij} = \beta_0 + \zeta_{(2)ij} + \zeta_{(3)j} + \epsilon_{ij}.$

Where $\hat{\theta}_{ij}$ is the estimation of the true effect size for public support, β_0 the average population effect, $\zeta_{(2)ij}$ is the within-country variance, $\zeta_{(3)j}$ is the between-country variance, and ϵ_{ij} the sampling variance.[34] In each model, heterogeneity will be quantified by the I^2 statistic per level. We intend to use R 3.6.5 (R Foundation for Statistical Computing, 2020) using the packages meta and metaphor for all analysis.[35,36] A second analysis will be performed on all studies presenting the change in public support following implementation of the actual policy under study. If relevant analyses will be performed twice, once including estimates for public support in general and once for public support specifically related to children, e.g. public support for smoking bans in cars and public support for smoking bans in cars if children are present (see section *outcomes and prioritisation)*.

For peer review only - http://bmjopen.bmj.com/site/about/guidelines.xhtml

The secondary objective is to identify and quantify determinants that are associated with public support. The determinants of public support will be evaluated at three levels:

1. Quantify within-study determinants of public support

Public support may differ between population subgroups. Therefore, we will conduct subgroup analyses according to:

- Gender (men vs. women)
- Smoking status (current smokers vs. former smokers vs. non-smokers, and/or current smokers vs. non-smokers (including former smokers), depending on data availability)
- Parental status (yes vs. no, depending on data availability)
- Age group (younger vs. older, categorisation depending on data availability)

Public support will be pooled per subgroup for each of the five spaces categories using random-effects three-level meta-analysis.[37]

2. Quantify between-study determinants of public support

Various study-specific elements may influence public support. Random-effects linear meta-regression analysis will be performed to assess between-study determinants of public support according to the various spaces that the policies cover . In these analyses support in favour of smoke-free policies is used as dependent variable and the following variables per study are used as independent variables:

- Calendar year in which the survey was conducted (continuous);
- Whether public support was assessed as yes-no or on a Likert-scale (binary);
- Income level of the country, according to the World Bank classification (binary: high- versus low- and middle-income countries); and
- Whether or not traditional-smoke-free legislation covering enclosed public places and workplaces was in place (categorical: none, partial, or comprehensive according to the WHO classification[23]).

3. Identify context-specific determinants of public support

Context-specific determinants of public support will be identified using thematic synthesis. We will follow the method outlined by Thomas and Harden consisting of three steps:[38] (1) coding text, (2) developing descriptive themes and (3) generating analytic themes. The full-text of each study will be extracted and uploaded into NVivo V.12 (NVivo Qualitative data analysis software V.10: QSR International Pty Ltd, 2012). As studies may provide information outside the scope of this review, coding will be limited to sentences describing details that relate to determinants of public support for smoke-free policies. In order to ensure a consistent coding methodology, three eligible articles will be coded independently by two reviewers and then compared until consensus on the themes has been reached. The remaining articles will be coded independently by two reviewers. After every five articles coding will be compared to ensure consistency. *A priori* four core domains have been identified: (1) beliefs and scientific evidence about effectiveness, (2) social norms, (3) communication and implementation strategies, and (4) collaboration between stakeholders.

For peer review only - http://bmjopen.bmj.com/site/about/guidelines.xhtml

Sensitivity analysis

Study findings may vary according to the risk-of-bias level of the individual studies. As a sensitivity analysis, we will exclude studies that scored *no* or *can't tell* on at least one domain following the MMAT criteria. This criterion is based on MMAT evaluations in previous literature.[39]

Ethics and dissemination

No primary data collection will be undertaken; therefore, no formal ethical assessment and informed consent are required. Findings will be summarised in a single manuscript and will be disseminated through scientific literature.

Timeline

Start date: April 15th 2020

Finishing date: 31 December 2020

Reporting date:

Authors contributions

JVB conceptualised the study and secured funding. All authors contributed to the design of the protocol. WMB developed the search strategy. NWB and FJMM wrote the first draft and revised subsequent drafts. JVB supervised the writing. AS, WMB, AB and FJVL contributed to the writing and have read and approved the final manuscript.

Funding statement

This work was funded by a joint collaboration between Lung Foundation Netherlands, Dutch Cancer Society, Dutch Heart Foundation, Dutch Diabetes Research Foundation and the Netherlands Thrombosis Foundation (grant number 2.1.19.010). AS is supported by Health Data Research UK.

Competing interests

The authors declare that they have no competing interests.

Patient and Public Involvement

Patient and public involvement Patients and/or the public were not involved in the design, or conduct, or reporting, or dissemination plans of this research.

References

1. Stanaway JD, Afshin A, Gakidou E, et al. Global, regional, and national comparative risk assessment of 84 behavioural, environmental and occupational, and metabolic risks or clusters of risks for 195 countries and territories, 1990–2017: a systematic analysis for the Global Burden of Disease Study 2017. *Lancet* 2018;**392**(10159):1923-94.
2. Hoffman SJ, Tan C. Overview of systematic reviews on the health-related effects of government tobacco control policies. *BMC Public Health* 2015;**15**:744.

For peer review only - http://bmjopen.bmj.com/site/about/guidelines.xhtml

For peer review only

3. Faber T, Kumar A, Mackenbach JP, et al. Effect of tobacco control policies on perinatal and child health: a systematic review and meta-analysis. *Lancet Public Health* 2017;**2**(9):e420-e37.
4. Frazer K, Callinan JE, McHugh J, et al. Legislative smoking bans for reducing harms from secondhand smoke exposure, smoking prevalence and tobacco consumption. *Cochrane Database Syst Rev* 2016;**2**:CD005992.
5. World Health Organization. WHO Report on the Global Tobacco Epidemic, 2009: implemeting smoke-free environments France: Geneva: World Health Organization, 2009.
6. Sureda X, Fernandez E, Lopez MJ, et al. Secondhand tobacco smoke exposure in open and semi-open settings: a systematic review. *Environ Health Perspect* 2013;**121**(7):766-73.
7. Licht AS, Hyland A, Travers MJ, et al. Secondhand smoke exposure levels in outdoor hospitality venues: a qualitative and quantitative review of the research literature. *Tob Control* 2013;**22**(3):172.
8. Allwright S. Republic of Ireland's indoor workplace smoking ban. *Br J Gen Pract* 2004;**54**(508):811-12.
9. Thomson G, Wilson N. Public attitudes to laws for smoke-free private vehicles: a brief review. *Tob Control* 2009;**18**(4):256-61.
10. Patel M, Thai CL, Meng Y-Y, et al. Smoke-free car legislation and student exposure to smoking. *Pediatrics* 2018;**141**(Supplement 1):S40-S50.
11. Faber T, Mizani MA, Sheikh A, et al. Investigating the effect of England's smoke-free private vehicle regulation on changes in tobacco smoke exposure and respiratory disease in children: a quasi-experimental study. *Lancet Public Health* 2019;**4**(12):e607-e17.
12. Sureda X, Ballbè M, Martínez C, et al. Impact of tobacco control policies in hospitals: Evaluation of a national smoke-free campus ban in Spain. *Prev Med Rep* 2014;**1**:56-61.
13. Fitzpatrick P, Gilroy I, Doherty K, et al. Implementation of a campus-wide Irish hospital smoking ban in 2009: prevalence and attitudinal trends among staff and patients in lead up. *Health Promot Int* 2009;**24**(3):211-22.
14. Johns M, Coady MH, Chan CA, et al. Evaluating New York City's smoke-free parks and beaches law: a critical multiplist approach to assessing behavioral impact. *Am J Community Psychol* 2013;**51**(1-2):254-63.
15. Levy DE, Adams IF, Adamkiewicz G. Delivering on the Promise of Smoke-Free Public Housing. *Am J Public Health* 2017;**107**(3):380-83.
16. International Agency for Research on Cancer World Health Organization. Evaluating the effectiveness of smoke-free policies. Lyon: IARC Press, International Agency for Research on Cancer, 2009.
17. Thomson G, Wilson N, Collins D, et al. Attitudes to smoke-free outdoor regulations in the USA and Canada: a review of 89 surveys. *Tob Control* 2016;**25**(5):506-16.
18. Fong GT, Hyland A, Borland R, et al. Reductions in tobacco smoke pollution and increases in support for smoke-free public places following the implementation of comprehensive smoke-free workplace legislation in the Republic of Ireland: findings from the ITC Ireland/UK Survey. *Tob Control* 2006;**15**(suppl 3):iii51.
19. Fabian LE, Bernat DH, Lenk KM, et al. Smoke-free laws in bars and restaurants: does support among teens and young adults change after a statewide smoke-free law? *Public Health Rep* 2011;**126**(5):669-76.
20. Thomson G, Wilson N. One year of smokefree bars and restaurants in New Zealand: impacts and responses. *BMC Public Health* 2006;**6**(1):64.
21. Kuijpers TG, Willemsen MC, Kunst AE. Public support for tobacco control policies: The role of the protection of children against tobacco. *Health Policy* 2018;**122**(8):929-35.

For peer review only

For peer review only - http://bmjopen.bmj.com/site/about/guidelines.xhtml

22. Rozema AD, Mathijssen JJP, Jansen MWJ, et al. Schools as smoke-free zones? Barriers and facilitators to the adoption of outdoor school ground smoking bans at secondary schools. *Tob Induc Dis* 2016;**14**(1):10.
23. World Health Organization. Protection from exposure to second-hand tobacco smoke: policy recommendations, 2007.
24. Cochran WG. Sampling Technique. John Wiley and Son. *New York* 1977.
25. Bartlett JE, Kotrlik JW, Higgins CCH. Organizational research: Determining appropriate sample size in survey research appropriate sample size in survey research. *Inf Technol J* 2001;**19**(1):8.
26. Vital Strategies. Crooked Nine: Nine Ways the Tobacco Industry Undermines Health Policy. New York, NY 2019.
27. Bramer WM. Reference checking for systematic reviews using Endnote. *J Med Libr Assoc* 2018;**106**(4):542-46.
28. Bramer WM, Giustini D, de Jonge GB, et al. De-duplication of database search results for systematic reviews in EndNote. *J Med Libr Assoc* 2016;**104**(3):240-3.
29. World Bank. Country Classification In: Group TWB, ed., 2019.
30. Hong QN, Pluye P, Fàbregues S, et al. Mixed methods appraisal tool (MMAT), version 2018. *IC Canadian Intellectual Property Office, Industry Canada* 2018.
31. Wang N. How to Conduct a Meta-Analysis of Proportions in R: A Comprehensive Tutorial, 2018.
32. Nyaga VN, Arbyn M, Aerts M. Metaprop: a Stata command to perform meta-analysis of binomial data. *Arch Public Health* 2014;**72**(1):39.
33. Van den Noortgate W, Lopez-Lopez JA, Marin-Martinez F, et al. Three-level meta-analysis of dependent effect sizes. *Behav Res Methods* 2013;**45**(2):576-94.
34. Assink M, Wibbelink CJM. Fitting three-level meta-analytic models in R: A step-by-step tutorial. *Quant Method Psychol* 2016;**12**(3):154-74.
35. Schwarzer G. meta: An R package for meta-analysis. *R news* 2007;**7**(3):40-45.
36. Viechtbauer W. Conducting meta-analyses in R with the metafor package. *J Stat Softw* 2010;**36**(3):1-48.
37. Borenstein M, Higgins JPT. Meta-analysis and subgroups. *Prev Sci* 2013;**14**(2):134-43.
38. Thomas J, Harden A. Methods for the thematic synthesis of qualitative research in systematic reviews. *BMC Med Res Methodol* 2008;**8**:45.
39. Eykelenboom M, van Stralen MM, Olthof MR, et al. Political and public acceptability of a sugar-sweetened beverages tax: a mixed-method systematic review and meta-analysis. *Int J Behav Nutr Phys Act* 2019;**16**(1):78.

For peer review only

For peer review only - http://bmjopen.bmj.com/site/about/guidelines.xhtml

Appendix I

embase.com (1974-)

('smoking regulation'/exp OR (smokefree OR ((smoking OR smoke OR tobacco) NEAR/3 (regulation* OR government* OR law OR laws OR policy OR policies OR ban OR bans OR banned OR free OR restrict* OR act OR acts))):ab,ti) AND ('public opinion'/exp OR 'public attitude'/de OR (opinion* OR support* OR views OR (public NEAR/3 view) OR attitude* OR feeling* OR acceptance* OR accepts OR perception* OR misperception*):ab,ti) NOT ([Letter]/lim OR [Note]/lim OR [Editorial]/lim)

Medline Ovid (1946-)

(Smoke-Free Policy/ OR (smokefree OR ((smoking OR smoke OR tobacco) ADJ3 (regulation* OR government* OR law OR laws OR policy OR policies OR ban OR bans OR banned OR free OR restrict* OR act OR acts))).ab,ti.) AND (Public Opinion/ OR Attitude/ OR (opinion* OR support* OR views OR (public ADJ3 view) OR attitude* OR feeling* OR acceptance* OR accepts OR perception* OR misperception*).ab,ti.) NOT (news OR book* OR chapter* OR dissertation abstract*).pt.

Web of science Core Collection (1975-)

TS=(((smokefree OR ((smoking OR smoke OR tobacco) NEAR/2 (regulation* OR government* OR law OR laws OR policy OR policies OR ban OR bans OR banned OR free OR restrict* OR act OR acts)))) AND ((opinion* OR support* OR views OR (public NEAR/2 view) OR attitude* OR feeling* OR acceptance* OR accepts OR perception* OR misperception*)))

PsycINFO Ovid (1806-)

((smokefree OR ((smoking OR smoke OR tobacco) ADJ3 (regulation* OR government* OR law OR laws OR policy OR policies OR ban OR bans OR banned OR free OR restrict* OR act OR acts))).ab,ti.) AND (Public Opinion/ OR Attitudes/ OR (opinion* OR support* OR views OR (public ADJ3 view) OR attitude* OR feeling* OR acceptance* OR accepts OR perception* OR misperception*).ab,ti.) NOT (news OR book* OR chapter* OR dissertation abstract*).pt.

LILACS

(smokefree OR smoking OR smoke) AND (opinions OR views OR attitudes)

Scientific Electronic Library Online [SciELO])

(smokefree OR smoking OR smoke) AND (opinions OR views OR attitudes)

WHO Global Health Library

(smokefree OR smoking OR smoke) AND (opinions OR views OR attitudes)

For peer review only

PRISMA-P (Preferred Reporting Items for Systematic review and Meta-Analysis Protocols) 2015 checklist: recommended items to address in a systematic review protocol*

Section and topic	Item No	Checklist item	Page
**ADMINISTRATIVE INFORMATION**			
Title:			
Identification	1a	Identify the report as a protocol of a systematic review	1
Update	1b	If the protocol is for an update of a previous systematic review, identify as such	N.A.
Registration	2	If registered, provide the name of the registry (such as PROSPERO) and registration number	2
Authors:			
Contact	3a	Provide name, institutional affiliation, e-mail address of all protocol authors; provide physical mailing address of corresponding author	1
Contributions	3b	Describe contributions of protocol authors and identify the guarantor of the review	10
Amendments	4	If the protocol represents an amendment of a previously completed or published protocol, identify as such and list changes; otherwise, state plan for documenting important protocol amendments	N.A.
Support:			
Sources	5a	Indicate sources of financial or other support for the review	10
Sponsor	5b	Provide name for the review funder and/or sponsor	10
Role of sponsor or funder	5c	Describe roles of funder(s), sponsor(s), and/or institution(s), if any, in developing the protocol	10
**INTRODUCTION**			
Rationale	6	Describe the rationale for the review in the context of what is already known	3
Objectives	7	Provide an explicit statement of the question(s) the review will address with reference to participants, interventions, comparators, and outcomes (PICO)	3
**METHODS**			
Eligibility criteria	8	Specify the study characteristics (such as PICO, study design, setting, time frame) and report characteristics (such as years considered, language, publication status) to be used as criteria for eligibility for the review	4
Information sources	9	Describe all intended information sources (such as electronic databases, contact with study authors, trial registers or other grey literature sources) with planned dates of coverage	5
Search strategy	10	Present draft of search strategy to be used for at least one electronic database, including planned limits, such that it could be repeated	Appendix I

For peer review only

Study records:			
Data management	11a	Describe the mechanism(s) that will be used to manage records and data throughout the review	5
Selection process	11b	State the process that will be used for selecting studies (such as two independent reviewers) through each phase of the review (that is, screening, eligibility and inclusion in meta-analysis)	6
Data collection process	11c	Describe planned method of extracting data from reports (such as piloting forms, done independently, in duplicate), any processes for obtaining and confirming data from investigators	6
Data items	12	List and define all variables for which data will be sought (such as PICO items, funding sources), any pre-planned data assumptions and simplifications	6
Outcomes and prioritization	13	List and define all outcomes for which data will be sought, including prioritization of main and additional outcomes, with rationale	7
Risk of bias in individual studies	14	Describe anticipated methods for assessing risk of bias of individual studies, including whether this will be done at the outcome or study level, or both; state how this information will be used in data synthesis	7
Data synthesis	15a	Describe criteria under which study data will be quantitatively synthesised	7
	15b	If data are appropriate for quantitative synthesis, describe planned summary measures, methods of handling data and methods of combining data from studies, including any planned exploration of consistency (such as I^2, Kendall's τ)	7
	15c	Describe any proposed additional analyses (such as sensitivity or subgroup analyses, meta-regression)	9
	15d	If quantitative synthesis is not appropriate, describe the type of summary planned	N.A.
Meta-bias(es)	16	Specify any planned assessment of meta-bias(es) (such as publication bias across studies, selective reporting within studies)	10
Confidence in cumulative evidence	17	Describe how the strength of the body of evidence will be assessed (such as GRADE)	-

*** It is strongly recommended that this checklist be read in conjunction with the PRISMA-P Explanation and Elaboration (cite when available) for important clarification on the items. Amendments to a review protocol should be tracked and dated. The copyright for PRISMA-P (including checklist) is held by the PRISMA-P Group and is distributed under a Creative Commons Attribution Licence 4.0.**

From: Shamseer L, Moher D, Clarke M, Ghersi D, Liberati A, Petticrew M, Shekelle P, Stewart L, PRISMA-P Group. Preferred reporting items for systematic review and meta-analysis protocols (PRISMA-P) 2015: elaboration and explanation. BMJ. 2015 Jan 2;349(jan02 1):g7647.

For peer review only

BMJ Open

Assessing public support for extending smoke-free policies beyond enclosed public places and workplaces: protocol for a systematic review and meta-analysis

Journal:	*BMJ Open*
Manuscript ID	bmjopen-2020-040167.R2
Article Type:	Protocol
Date Submitted by the Author:	23-Nov-2020
Complete List of Authors:	Boderie, Nienke; Erasmus MC, University Medical Centre Rotterdam, Department of Public Health<br>Mölenberg, Famke; Erasmus MC, University Medical Centre Rotterdam, Department of Public Health<br>Sheikh, Aziz; University of Edinburgh, Centre of Medical Informatics, Usher Institute ; Brigham and Women's Hospital/Harvard Medical School, Division of General Internal Medicine and Primary Care<br>Bramer, Wichor; Erasmus MC, University Medical Centre Rotterdam, Medical Library<br>Burdorf, Alex; Erasmus MC, University Medical Centre Rotterdam, Department of Public Health<br>Van Lenthe, Frank; Erasmus MC, University Medical Centre Rotterdam, Department of Public Health<br>Been, Jasper; Erasmus MC, University Medical Centre Rotterdam, Department of Public Health; Erasmus MC - Sophia Children’s Hospital, University Medical Centre Rotterdam, Department of Paediatrics
**Primary Subject Heading**:	Public health
Secondary Subject Heading:	Health policy, Public health
Keywords:	PUBLIC HEALTH, PREVENTIVE MEDICINE, Health policy < HEALTH SERVICES ADMINISTRATION & MANAGEMENT

SCHOLARONE™
Manuscripts

I, the Submitting Author has the right to grant and does grant on behalf of all authors of the Work (as defined in the below author licence), an exclusive licence and/or a non-exclusive licence for contributions from authors who are: i) UK Crown employees; ii) where BMJ has agreed a CC-BY licence shall apply, and/or iii) in accordance with the terms applicable for US Federal Government officers or employees acting as part of their official duties; on a worldwide, perpetual, irrevocable, royalty-free basis to BMJ Publishing Group Ltd ("BMJ") its licensees and where the relevant Journal is co-owned by BMJ to the co-owners of the Journal, to publish the Work in this journal and any other BMJ products and to exploit all rights, as set out in our licence.

The Submitting Author accepts and understands that any supply made under these terms is made by BMJ to the Submitting Author unless you are acting as an employee on behalf of your employer or a postgraduate student of an affiliated institution which is paying any applicable article publishing charge ("APC") for Open Access articles. Where the Submitting Author wishes to make the Work available on an Open Access basis (and intends to pay the relevant APC), the terms of reuse of such Open Access shall be governed by a Creative Commons licence – details of these licences and which Creative Commons licence will apply to this Work are set out in our licence referred to above.

Other than as permitted in any relevant BMJ Author's Self Archiving Policies, I confirm this Work has not been accepted for publication elsewhere, is not being considered for publication elsewhere and does not duplicate material already published. I confirm all authors consent to publication of this Work and authorise the granting of this licence.

For peer review only

Assessing public support for extending smoke-free policies beyond enclosed public places and workplaces: protocol for a systematic review and meta-analysis

Registration number …

Nienke W. Boderie[1], Famke J.M. Mölenberg[1], Aziz Sheikh[2,3], Wichor M. Bramer[4], Alex Burdorf[1], Frank J. van Lenthe[1], Jasper V. Been[1,2,5]

[1] Department of Public Health, Erasmus MC, University Medical Centre Rotterdam, Rotterdam, Netherlands

[2] Centre of Medical Informatics, Usher Institute, The University of Edinburgh, Edinburgh, UK

[3] Division of General Internal Medicine and Primary Care, Brigham and Women's Hospital/Harvard Medical School, Boston, MA, USA

[4] Medical Library, Erasmus MC, University Medical Centre Rotterdam, Rotterdam, Netherlands

[5] Department of Paediatrics, division of Neonatology, Erasmus MC - Sophia Children's Hospital, University Medical Centre Rotterdam, Rotterdam, Netherlands

Corresponding author:
Jasper V. Been
Erasmus University Medical Centre, PO Box 2040, 3000 CA Rotterdam
j.been@erasmusmc.nl
+31 10 70 36077

Word count: 3417

For peer review only

For peer review only - http://bmjopen.bmj.com/site/about/guidelines.xhtml

KEYWORDS: Smoke-Free Policy, Tobacco Smoke Pollution, Attitude

ABSTRACT: (335 words)

Introduction: Smoke-free enclosed public environments are effective in reducing exposure to second-hand smoke and yield major public health benefits. Building on this, many countries are now implementing smoke-free policies regulating smoking beyond enclosed public places and workplaces. In order to successfully implement such 'novel smoke-free policies' public support is essential. We aim to provide the first comprehensive systematic review and meta-analysis assessing levels and determinants of public support for novel smoke-free policies.

Methods and analysis: The primary objective of this review is to summarise the level of public support for novel smoke-free policies. Eight online databases (Embase.com, Medline ALL Ovid, Web of Science Core Collection, WHO Library Database, Latin American and Caribbean Health Sciences Literature, Scientific Online Library Online, PsychINFO, and Google Scholar) will be searchedfrom 1 January 2004 by two independent researchers with no language restrictions. The initial search was performed on April 15 2020 and will be updated prior to finalisation of the report. Studies are eligible if assessing support for novel smoke-free policies in the general population (age ≥16 years) and have a sample size of n≥400. Studies funded by the tobacco industry or evaluating support among groups with vested interest are excluded. The primary outcome is proportion of public support for smoke-free policies, subdivided according to the spaces covered: (1) indoor private spaces (e.g. cars), (2) indoor semi-private spaces (e.g. multi-unit housing), (3) outdoor (semi-)private spaces (e.g. courtyards), (4) non-hospitality outdoor public spaces (e.g. parks, hospital grounds, playgrounds), and (5) hospitality outdoor public spaces (e.g. restaurant terraces).The secondary objective is to identify determinants associated with public support on three levels: (1) within-study determinants (e.g. smoking status), (2) between-study determinants (e.g. survey year), and (3) context-specific determinants (e.g. social norms). Risk of bias will be assessed using the Mixed Methods Appraisal Tool and a sensitivity analysis will be performed excluding studies at high-risk-of-bias.

Ethics and dissemination: No formal ethical approval is required. Findings will be disseminated to academics, policy-makers and the general public.

The protocol is registered with PROSPERO registration number: ?

STRENGHTS AND LIMITATIONS

- This systematic review is unique in providing a structured overview of levels of public support for 'novel smoke-free policies' (i.e. smoke free policies that go beyond regulating smoking in enclosed public places and workplaces)
- Within- and between-study determinants associated with public support will be assessed, and thematic synthesis will be used to identify context-specific determinants.
- The protocol presented has been designed in line with the Preferred Reporting Items for Systematic Reviews and Meta-Analysis for Protocols (PRISMA-P)
- The generalizability and value of this systematic review depends on the availability and quality of the data.

For peer review only - http://bmjopen.bmj.com/site/about/guidelines.xhtml

INTRODUCTION

Second-hand smoke (SHS) exposure is related to 1.2 million deaths per year.[1] Smoke-free environments have proven to be effective in reducing exposure to SHS and have major public health benefits.[2] Previous systematic reviews reported consistent evidence for improved cardiovascular health and reduced smoking-related mortality, as well as reductions in preterm birth, severe asthma exacerbations and respiratory tract infections in children, following implementation of smoke-free legislation in indoor public places and workplaces.[3-5] It has been shown that outdoor areas contribute significantly to SHS exposure, therefore the implementation of smoking-free policies in open spaces have the potential to reduce the associated burden of disease.[6,7]
In 2004, Ireland was the first country in the world to implement comprehensive smoke-free legislation covering enclosed workplaces and public places, and many more countries followed its example.[8] An increasing number of jurisdictions is now implementing, or considering implementing, additional smoke-free policies that go beyond regulating smoking in enclosed public places and target private and outdoor spaces, henceforth referred to as 'novel smoke-free policies'. Novel smoke-free policies are implemented in an attempt to further improve population health via reducing SHS exposure. For example, several countries have implemented laws requiring private cars carrying children be smoke-free,[9-11] smoke free hospital campuses have been implemented country wide in Spain and Ireland,[12,13] the city of New York banned smoking in all public parks, pedestrian plazas and at all beaches,[14] and the US Department of Public Housing and Urban Development requires all public housing units to be smoke-free, both within resident units and in public areas.[15] Public support is essential in democracies in order for policy-makers to consider implementing such novel smoke-free policies and to increase the likelihood of successful implementation,[16] and accordingly the World Health Organisation (WHO) stated that "Involving civil society is central to achieving effective legislation".[5]

However, public support may vary over time, as well as by population subgroups. For example, women and non-smokers tend to be more in favour of smoke-free legislation than men and current smokers.[17] Several studies showed that public support for smoke-free policies increased after successful implementation and particularly so among smokers.[18-20] Furthermore, public support for smoke-free policies was higher when policies covered spaces that were frequently visited by those more vulnerable to the adverse health effects of SHS.[21] For example, in the US and Canada public support for smoke-free playgrounds (89-91%) was substantially higher than for smoke-free outdoor workplaces (12-46%) and sidewalks (31-49%).[17] Context-specific determinants may also contribute to differences in public support across settings. Aspects that enhanced successful adoption of smoke-free zones at outdoor school grounds at secondary schools included communication about the policy, collaboration between and within stakeholders, social norms, and evidence about the effectiveness of smoke-free zones.[22]

A structured overview of the levels and determinants of public support for smoke-free policies beyond enclosed public places and workplaces across various settings is currently lacking. Having these insights may guide policy makers with the implementation of policies that receive the highest levels of support, and may help in defining additional strategies that are needed to increase public support in the population. To address this gap in the literature our primary objective is to summarise the level of public support across the globe for novel smoke-free policies and to evaluate if public support changed following implementation of the novel smoke-free policies across various settings. To do so a systematic review and meta-analysis will be conducted. The secondary objective is to identify determinants associated with public support at the following three levels: 1) within-study determinants (e.g. age,

For peer review only - http://bmjopen.bmj.com/site/about/guidelines.xhtml

smoking status, parental status), 2) between-study determinants (e.g. income level of the country, whether smoke-free legislation in enclosed public places and workplaces was already in place), and 3) context-specific determinants (e.g. setting, framing, enforcement of smoke-free policies).

METHODS AND ANALYSIS

We used the Preferred Reporting Items for Systematic Reviews and Meta-Analysis Protocols (PRISMA-P) guidelines to facilitate development of this protocol, see appendix I. The protocol has been registered in PROSPERO as ?

In this review, we will use the term 'traditional smoke-free legislation' to refer to smoke-free legislation covering enclosed public places and workplaces (i.e. compliant with Article 8(2) of the Framework Convention on Tobacco Control; FCTC) and the term 'novel smoke-free policies' to refer to policies and legislation regulating smoking in any other places, such as (semi)private places and (partially) outdoor spaces, whether public or (semi)private.[23] Policies are used in the broadest sense and are not necessarily enacted via formal legislation as this will allow us to evaluate less formal local smoke-free initiatives (e.g. self-regulation by the hospitality sector or local hospitals) as well as formal legislation.

Eligibility criteria

We will include articles published in scientific journals as well as 'grey literature' evaluating public support for novel smoke-free policies covering (semi)private places and (partially) outdoor spaces, whether public or (semi)private. Grey literature includes policy documents and reports that are published non-commercially and/or are not indexed by major scientific literature databases. Cohort studies and (repeated) cross-sectional studies will be included and no language restrictions are applied. Qualitative studies will be excluded. We will seek translation for reports in foreign languages to assess eligibility. Studies for which only an abstract is available, will not be included since risk of bias for these studies cannot be adequately assessed.

Eligibility of the studies will be assessed using the following criteria:

(1) Studies will be eligible if support for one or more novel smoke-free policies is evaluated. We will include studies assessing support for novel smoke-free policies that are already in place as well as those assessing support for upcoming or theoretical implementation of such policies. Policies at any level are eligible, such as city-level, state-level, country level etc. Studies will be excluded if solely evaluating traditional smoke-free legislation.[23]

(2) Studies will be eligible if they assessed public support for smoke-free policies in the population aged 16 years or above who represent the majority of a population primarily affected by the policy (e.g. support for a country wide measure is evaluated in a representative sample of the country, while support for a policy at a local campus is assessed among students and staff of that specific campus), or in any of the predefined population subgroups (see *within-study determinants of public support* below). We set this age criterion to include the part of the population that is entitled to vote in most democracies, and as such may be regarded to be of particular interest to politicians and policymakers. Any study reporting (sub)populations in which at least 50% fits this age criterion will also be included.

(3) Our primary objective is to summarise the level of public support for novel smoke free policies in the general population, therefore we will only include studies of which we can be confident

For peer review only - http://bmjopen.bmj.com/site/about/guidelines.xhtml

that the reported support in the study sample reflects the levels of support that would be found if the entire population was surveyed. This is operationalized by only including studies can ensure a 5% margin of error. Following sample size calculations for surveys, [24,25] a minimum sample size of 400 is required. A similar criterion was used by an earlier review assessing public support for outdoor smoke-free areas.[17]

(4) Studies will be included when published from 1 Jan 2004 onwards. This pragmatic cut-off was chosen as the first national traditional smoke-free law covering indoor public places and workplaces was introduced in Ireland in 2004. Hence, assessments of public support for novel smoke-free policies are unlikely to have preceded 2004, and are unlikely to be relevant for current everyday practice if they have.

(5) Studies will be excluded if solely evaluating support among specific subgroups not representing the majority of the population primarily affected by the policy, policy makers or groups with clearly vested interest, e.g. opinion of tobacco industry groups.

(6) Studies will be excluded when funded or supported by the tobacco industry, as the tobacco industry is known to "produce, sponsor and disseminate misleading research and information, lacking sound scientific methods". [26]

(7) Studies will be excluded if solely evaluating support for tobacco related subgroups, e.g. e-cigarettes or heatless tobacco products.

Information sources

The following electronic databases will be searched for eligible studies: (1) embase.com, (2) Medline ALL Ovid, (3) Web of Science Core Collection, (4) WHO Library Database (WHOLIS), (5) Latin American and Caribbean Health Sciences Literature (LILACS), (6) Scientific Online Library Online (SciELO), (7) PsychINFO and (8) Google Scholar.

Search strategy

The specific search strategies per database have been created in close collaboration with a bibliographic expert of the Erasmus MC with expertise in systematic reviewing (WMB; see Appendix 1). Search terms include three parts: (1) terms to identify smoke-free policies; (2) terms to identify measures of public support as the outcome; (3) terms that exclude letters to the editors, notes and editorials; and (4) terms to exclude items published prior to 1 January 2004.

We will complement our search by screening reference lists of reviews related to the topic and of included studies and their citations through Scopus, following Bramer.[27] We will update our search to add the most recent reports just before submitting our final review report for publication.

Study records

Data management

All records identified by the search strategy will be extracted into an Endnote Library, and we will de-duplicate using this software following the procedure outlined by Bramer. [28] If any duplicates remain those will be manually excluded. At this stage, duplicates will be identified based on overlapping author names and titles. The total number of detected duplicates will be noted in the final report.

For peer review only - http://bmjopen.bmj.com/site/about/guidelines.xhtml

Selection process

After removing duplicates, titles and abstracts of records identified during the literature search will be screened independently for inclusion by two reviewers. After initial selection based on screening of titles and abstracts, full-text articles will be screened for eligibility according to the inclusion and exclusion criteria by two reviewers, and discrepancies will be resolved after discussion with a third reviewer. Remaining duplicates based on populations, sample size and reported outcomes will be identified based on full text. The reviewers will not be blinded to information about the articles (e.g. authors' names and affiliations) at any stage.

Data collection process

Two reviewers will independently extract relevant data from all included studies according to a customised data extraction form developed a *priori* that was piloted using four eligible studies. Upon completion the reviewers will compare their results and any discrepancies will again be resolved after discussion with a third reviewer. If any relevant data are missing the corresponding authors will be contacted.

Data items

Customised data extraction forms will be used to extract relevant information from the eligible studies, which will include the following items:

(1) First author's name and affiliation
(2) Publication year
(3) Type of publication
(4) Access information (DOI or URL)
(5) Study design
(6) Location of the study (e.g. country, region)
(7) Description of the policy (e.g. places covered, whether or not the intervention is implemented, national or regional/local implementation)
(8) For studies assessing support for policies already implemented:
    a) date of implementation,
    b) level of implementation (e.g. government, municipality),
    c) level of enforcement (e.g. voluntary, warnings, fines)
(9) Observational period
(10) Selection of participants (e.g. eligibility criteria, sampling methods)
(11) Number of participants
(12) Data source (e.g. national survey, study recruited participants)
(13) Method of data collection
(14) Definition of public support
(15) Statistical analyses (if applicable)
(16) Number and percentages of missing values and non-response (if applicable)
(17) Techniques for handling missing values and non-response
(18) Characteristics of the study population (e.g. age, gender, smoking status)
(19) Levels of public support (estimate, 95% confidence intervals)
(20) Determinants of public support (see section *data synthesis* for more detail)

For peer review only - http://bmjopen.bmj.com/site/about/guidelines.xhtml

a) Within-study determinants
b) Between-study determinants
c) Context-specific determinants

(21) Any conflict of interest reported by the authors
(22) Funding source(s)

Data will be complemented with the World Bank Country Classification by income, based on Gross National Income (GNI) per capita.[29] Furthermore, we will seek information regarding whether at the time of the study traditional smoke-free regulation was already implemented in enclosed public areas and workspaces according to the WHO.[23]

Outcomes and prioritisation

Data will be extracted for each estimate of public support by the spaces that they cover (e.g. playgrounds, private cars, multi-unit housing). If weighted and unweighted estimates are presented, we will extract estimates that are weighted to most adequately reflect the general population. If multiple estimates are presented that relate to public support, we will extract the estimate that covers the most general spaces. For example, we will prioritise “it should be illegal to smoke in all playgrounds” above “it should be illegal to smoke in this specific playground”. If public support is asked in general and specifically related to children, we will extract both estimates. For example, we will extract “it should be illegal to smoke in private cars” and “it should be illegal to smoke in private cars when minors are present”.

Risk of bias assessment

We will assess risk of bias for each study using the Mixed Methods Appraisal Tool (MMAT) for descriptive studies. The MMAT 2018 version was developed based on criteria from 18 existing critical appraisal tools and input from over 50 international experts. The following five elements will be assessed: relevance of the sampling strategy, representativeness of the target population, appropriateness of the outcome measurements, risk of non-response bias, and appropriateness of the statistical techniques. Each of the elements will be categorised by using the answer categories *yes*, *no* or *can’t tell*, following MMAT criteria. Results of the risk-of-bias analysis will be presented in tables.[30]

Data synthesis

Obtaining comparable data is essential to facilitate meta-analysis, thus homogenisation of the outcome data is needed. Public support will be analysed as proportional data, i.e. proportion of the population supporting a particular smoke-free policy. When results are fairly normal distributed the raw proportions will be analysed, if not logit transformations will be applied[31]The outcome estimates will be reversed if studies report on the proportion not in favour of the smoke-free policies. Often Likert-scale type questions are used to assess support, if studies report percentages per answer option instead of total support, the answer categories above neutral (i.e. indicating a positive response) will be combined.

To allow meta-analyses, standard errors (SE) are needed. If SE are not presented they will be calculated using the following formula;[32]

$$fraction = \frac{nr\ of\ people\ supporting\ policy}{sample\ size}$$

For peer review only - http://bmjopen.bmj.com/site/about/guidelines.xhtml

$$SE = \sqrt{\frac{fraction(1 - fraction)}{sample\ size}}$$

Two reviewers will independently assess whether measures of public support and smoke-free policies under investigation are sufficiently comparable across the selected studies to allow meta-analysis. If needed, they will convert the units of measurement in a way that is consistent across studies. In case of disagreement, a third reviewer will decide which measures to use.

Prior to undertaking meta-analyses, we will subdivide policies by spaces that they cover according to the following division: (1) indoor private spaces (e.g. cars), (2) indoor semi-private spaces (e.g. multi-unit housing), (3) outdoor (semi-)private spaces (e.g. courtyard, psychiatric hospital), (4) non-hospitality outdoor public spaces (e.g. parks, streets, beaches, hospital grounds, playgrounds), and (5) hospitality outdoor public spaces (e.g. restaurant terraces). Separate meta-analyses will be conducted to assess public support for smoke-free policies according to these categories. If multiple estimates of public support are presented that cover similar spaces according to our categorisation, we will calculate the average public support across these spaces for use in meta-analyses. Thus, if studies present separate estimates of public support for playgrounds, parks and beaches (all belonging to the category 'non-hospitality outdoor public spaces'), the average of the three will be used. In case of overlapping study samples, we will include the study or effect estimation that: (1) uses national surveys and therefore is likely to be representative of the general population, (2) has the lowest risk of bias, or (3) incorporates the largest sample size, following this hierarchy. We aim to summarise the most up-to-date status of public support for novel smoke-free policies; thus, we will include the most recent estimation if studies presented multiple estimates over time. In secondary analyses we will evaluate whether public support changed following the actual introduction of the smoke-free policy under study, if the data allows.

One of the assumptions in meta-analysis is that effect sizes are independent, i.e. the effect size of one study does not imply the direction or magnitude of the effect size in another study.[33] Multiple estimates of public support in a specific country may violate the independence assumption, therefore we will conduct a three-level meta-analysis.[34] A three-level meta-analysis is an extended version of a random-effects meta-analysis and includes sampling variation at the first level, within-country heterogeneity at the second level and between-country heterogeneity at the third level. The analytic model is as follows:

(1) $\hat{\theta}_{ij} = \beta_0 + \zeta_{(2)ij} + \zeta_{(3)j} + \epsilon_{ij}$.

Where $\hat{\theta}_{ij}$is the estimation of the true effect size for public support, β_0 the average population effect, $\zeta_{(2)ij}$ is the within-country variance, $\zeta_{(3)j}$ is the between-country variance, and ϵ_{ij} the sampling variance. [34] In each model, heterogeneity will be quantified by the I^2 statistic per level. We intend to use R 3.6.5 (R Foundation for Statistical Computing, 2020) using the packages meta and metaphor for all analysis. [35,36] A second analysis will be performed on all studies presenting the change in public support following implementation of the actual policy under study. If relevant analyses will be performed twice, once including estimates for public support in general and once for public support specifically related to children, e.g. public support for smoking bans in cars and public support for smoking bans in cars if children are present (see section *outcomes and prioritisation)*.

For peer review only - http://bmjopen.bmj.com/site/about/guidelines.xhtml

The secondary objective is to identify and quantify determinants that are associated with public support. The determinants of public support will be evaluated at three levels:

1. Quantify within-study determinants of public support

Public support may differ between population subgroups. Therefore, we will conduct subgroup analyses according to:

- Gender (men vs. women)
- Smoking status (current smokers vs. former smokers vs. non-smokers, and/or current smokers vs. non-smokers (including former smokers), depending on data availability)
- Parental status (yes vs. no, depending on data availability)
- Age group (younger vs. older, categorisation depending on data availability)

Public support will be pooled per subgroup for each of the five spaces categories using random-effects three-level meta-analysis.[37]

2. Quantify between-study determinants of public support

Various study-specific elements may influence public support. Random-effects linear meta-regression analysis will be performed to assess between-study determinants of public support according to the various spaces that the policies cover. In these analyses support in favour of smoke-free policies is used as dependent variable and the following variables per study are used as independent variables:

- Calendar year in which the survey was conducted (continuous);
- Whether public support was assessed as yes-no or on a Likert-scale (binary);
- Income level of the country, according to the World Bank classification (binary: high- versus low- and middle-income countries); and
- Whether or not traditional-smoke-free legislation covering enclosed public places and workplaces was in place (categorical: none, partial, or comprehensive according to the WHO classification).[23]

3. Identify context-specific determinants of public support

Context-specific determinants of public support will be identified using thematic synthesis. We will follow the method outlined by Thomas and Harden consisting of three steps:[38] (1) coding text, (2) developing descriptive themes and (3) generating analytic themes. The full-text of each study will be extracted and uploaded into NVivo V.12 (NVivo Qualitative data analysis software V.10: QSR International Pty Ltd, 2012). As studies may provide information outside the scope of this review, coding will be limited to sentences describing details that relate to determinants of public support for smoke-free policies. In order to ensure a consistent coding methodology, three eligible articles will be coded independently by two reviewers and then compared until consensus on the themes has been reached. The remaining articles will be coded independently by two reviewers. After every five articles coding will be compared to ensure consistency. *A priori* four core domains have been identified: (1) beliefs and scientific evidence about effectiveness, (2) social norms, (3) communication and implementation strategies, and (4) collaboration between stakeholders.

For peer review only - http://bmjopen.bmj.com/site/about/guidelines.xhtml

Sensitivity analysis

Study findings may vary according to the risk-of-bias level of the individual studies. As a sensitivity analysis, we will exclude studies that scored *no* or *can't tell* on at least one domain following the MMAT criteria. This criterion is based on MMAT evaluations in previous literature.[39]

Ethics and dissemination

No primary data collection will be undertaken; therefore, no formal ethical assessment and informed consent are required. Findings will be summarised in a single manuscript and will be disseminated through scientific literature.

Timeline

Start date: April 15th 2020

Finishing date: 31 December 2020

Reporting date:

Authors contributions

JVB conceptualised the study and secured funding. All authors contributed to the design of the protocol. WMB developed the search strategy. NWB and FJMM wrote the first draft and revised subsequent drafts. JVB supervised the writing. AS, WMB, AB and FJVL contributed to the writing and have read and approved the final manuscript.

Funding statement

This work was funded by a joint collaboration between Lung Foundation Netherlands, Dutch Cancer Society, Dutch Heart Foundation, Dutch Diabetes Research Foundation and the Netherlands Thrombosis Foundation (grant number 2.1.19.010). AS is supported by Health Data Research UK.

Competing interests

The authors declare that they have no competing interests.

Patient and Public Involvement

Patient and public involvement Patients and/or the public were not involved in the design, or conduct, or reporting, or dissemination plans of this research.

References

1. Stanaway JD, Afshin A, Gakidou E, et al. Global, regional, and national comparative risk assessment of 84 behavioural, environmental and occupational, and metabolic risks or clusters of risks for 195 countries and territories, 1990–2017: a systematic analysis for the Global Burden of Disease Study 2017. *Lancet* 2018;**392**(10159):1923-94.
2. Hoffman SJ, Tan C. Overview of systematic reviews on the health-related effects of government tobacco control policies. *BMC Public Health* 2015;**15**:744.

For peer review only - http://bmjopen.bmj.com/site/about/guidelines.xhtml

3. Faber T, Kumar A, Mackenbach JP, et al. Effect of tobacco control policies on perinatal and child health: a systematic review and meta-analysis. *Lancet Public Health* 2017;**2**(9):e420-e37.
4. Frazer K, Callinan JE, McHugh J, et al. Legislative smoking bans for reducing harms from secondhand smoke exposure, smoking prevalence and tobacco consumption. *Cochrane Database Syst Rev* 2016;**2**:CD005992.
5. World Health Organization. WHO Report on the Global Tobacco Epidemic, 2009: implemeting smoke-free environments France: Geneva: World Health Organization, 2009.
6. Sureda X, Fernandez E, Lopez MJ, et al. Secondhand tobacco smoke exposure in open and semi-open settings: a systematic review. *Environ Health Perspect* 2013;**121**(7):766-73.
7. Licht AS, Hyland A, Travers MJ, et al. Secondhand smoke exposure levels in outdoor hospitality venues: a qualitative and quantitative review of the research literature. *Tob Control* 2013;**22**(3):172.
8. Allwright S. Republic of Ireland's indoor workplace smoking ban. *Br J Gen Pract* 2004;**54**(508):811-12.
9. Thomson G, Wilson N. Public attitudes to laws for smoke-free private vehicles: a brief review. *Tob Control* 2009;**18**(4):256-61.
10. Patel M, Thai CL, Meng Y-Y, et al. Smoke-free car legislation and student exposure to smoking. *Pediatrics* 2018;**141**(Supplement 1):S40-S50.
11. Faber T, Mizani MA, Sheikh A, et al. Investigating the effect of England's smoke-free private vehicle regulation on changes in tobacco smoke exposure and respiratory disease in children: a quasi-experimental study. *Lancet Public Health* 2019;**4**(12):e607-e17.
12. Sureda X, Ballbè M, Martínez C, et al. Impact of tobacco control policies in hospitals: Evaluation of a national smoke-free campus ban in Spain. *Prev Med Rep* 2014;**1**:56-61.
13. Fitzpatrick P, Gilroy I, Doherty K, et al. Implementation of a campus-wide Irish hospital smoking ban in 2009: prevalence and attitudinal trends among staff and patients in lead up. *Health Promot Int* 2009;**24**(3):211-22.
14. Johns M, Coady MH, Chan CA, et al. Evaluating New York City's smoke-free parks and beaches law: a critical multiplist approach to assessing behavioral impact. *Am J Community Psychol* 2013;**51**(1-2):254-63.
15. Levy DE, Adams IF, Adamkiewicz G. Delivering on the Promise of Smoke-Free Public Housing. *Am J Public Health* 2017;**107**(3):380-83.
16. International Agency for Research on Cancer World Health Organization. Evaluating the effectiveness of smoke-free policies. Lyon: IARC Press, International Agency for Research on Cancer, 2009.
17. Thomson G, Wilson N, Collins D, et al. Attitudes to smoke-free outdoor regulations in the USA and Canada: a review of 89 surveys. *Tob Control* 2016;**25**(5):506-16.
18. Fong GT, Hyland A, Borland R, et al. Reductions in tobacco smoke pollution and increases in support for smoke-free public places following the implementation of comprehensive smoke-free workplace legislation in the Republic of Ireland: findings from the ITC Ireland/UK Survey. *Tob Control* 2006;**15**(suppl 3):iii51.
19. Fabian LE, Bernat DH, Lenk KM, et al. Smoke-free laws in bars and restaurants: does support among teens and young adults change after a statewide smoke-free law? *Public Health Rep* 2011;**126**(5):669-76.
20. Thomson G, Wilson N. One year of smokefree bars and restaurants in New Zealand: impacts and responses. *BMC Public Health* 2006;**6**(1):64.
21. Kuijpers TG, Willemsen MC, Kunst AE. Public support for tobacco control policies: The role of the protection of children against tobacco. *Health Policy* 2018;**122**(8):929-35.

For peer review only - http://bmjopen.bmj.com/site/about/guidelines.xhtml

22. Rozema AD, Mathijssen JJP, Jansen MWJ, et al. Schools as smoke-free zones? Barriers and facilitators to the adoption of outdoor school ground smoking bans at secondary schools. *Tob Induc Dis* 2016;**14**(1):10.
23. World Health Organization. Protection from exposure to second-hand tobacco smoke: policy recommendations, 2007.
24. Cochran WG. Sampling Technique. John Wiley and Son. *New York* 1977.
25. Bartlett JE, Kotrlik JW, Higgins CCH. Organizational research: Determining appropriate sample size in survey research appropriate sample size in survey research. *Inf Technol J* 2001;**19**(1):8.
26. Vital Strategies. Crooked Nine: Nine Ways the Tobacco Industry Undermines Health Policy. New York, NY 2019.
27. Bramer WM. Reference checking for systematic reviews using Endnote. *J Med Libr Assoc* 2018;**106**(4):542-46.
28. Bramer WM, Giustini D, de Jonge GB, et al. De-duplication of database search results for systematic reviews in EndNote. *J Med Libr Assoc* 2016;**104**(3):240-3.
29. World Bank. Country Classification In: Group TWB, ed., 2019.
30. Hong QN, Pluye P, Fàbregues S, et al. Mixed methods appraisal tool (MMAT), version 2018. *IC Canadian Intellectual Property Office, Industry Canada* 2018.
31. Wang N. How to Conduct a Meta-Analysis of Proportions in R: A Comprehensive Tutorial, 2018.
32. Nyaga VN, Arbyn M, Aerts M. Metaprop: a Stata command to perform meta-analysis of binomial data. *Arch Public Health* 2014;**72**(1):39.
33. Van den Noortgate W, Lopez-Lopez JA, Marin-Martinez F, et al. Three-level meta-analysis of dependent effect sizes. *Behav Res Methods* 2013;**45**(2):576-94.
34. Assink M, Wibbelink CJM. Fitting three-level meta-analytic models in R: A step-by-step tutorial. *Quant Method Psychol* 2016;**12**(3):154-74.
35. Schwarzer G. meta: An R package for meta-analysis. *R news* 2007;**7**(3):40-45.
36. Viechtbauer W. Conducting meta-analyses in R with the metafor package. *J Stat Softw* 2010;**36**(3):1-48.
37. Borenstein M, Higgins JPT. Meta-analysis and subgroups. *Prev Sci* 2013;**14**(2):134-43.
38. Thomas J, Harden A. Methods for the thematic synthesis of qualitative research in systematic reviews. *BMC Med Res Methodol* 2008;**8**:45.
39. Eykelenboom M, van Stralen MM, Olthof MR, et al. Political and public acceptability of a sugar-sweetened beverages tax: a mixed-method systematic review and meta-analysis. *Int J Behav Nutr Phys Act* 2019;**16**(1):78.

For peer review only

For peer review only - http://bmjopen.bmj.com/site/about/guidelines.xhtml

Appendix I

embase.com (1974-)

('smoking regulation'/exp OR (smokefree OR ((smoking OR smoke OR tobacco) NEAR/3 (regulation* OR government* OR law OR laws OR policy OR policies OR ban OR bans OR banned OR free OR restrict* OR act OR acts))):ab,ti) AND ('public opinion'/exp OR 'public attitude'/de OR (opinion* OR support* OR views OR (public NEAR/3 view) OR attitude* OR feeling* OR acceptance* OR accepts OR perception* OR misperception*):ab,ti) NOT ([Letter]/lim OR [Note]/lim OR [Editorial]/lim)

Medline Ovid (1946-)

(Smoke-Free Policy/ OR (smokefree OR ((smoking OR smoke OR tobacco) ADJ3 (regulation* OR government* OR law OR laws OR policy OR policies OR ban OR bans OR banned OR free OR restrict* OR act OR acts))).ab,ti.) AND (Public Opinion/ OR Attitude/ OR (opinion* OR support* OR views OR (public ADJ3 view) OR attitude* OR feeling* OR acceptance* OR accepts OR perception* OR misperception*).ab,ti.) NOT (news OR book* OR chapter* OR dissertation abstract*).pt.

Web of science Core Collection (1975-)

TS=(((smokefree OR ((smoking OR smoke OR tobacco) NEAR/2 (regulation* OR government* OR law OR laws OR policy OR policies OR ban OR bans OR banned OR free OR restrict* OR act OR acts)))) AND ((opinion* OR support* OR views OR (public NEAR/2 view) OR attitude* OR feeling* OR acceptance* OR accepts OR perception* OR misperception*)))

PsycINFO Ovid (1806-)

((smokefree OR ((smoking OR smoke OR tobacco) ADJ3 (regulation* OR government* OR law OR laws OR policy OR policies OR ban OR bans OR banned OR free OR restrict* OR act OR acts))).ab,ti.) AND (Public Opinion/ OR Attitudes/ OR (opinion* OR support* OR views OR (public ADJ3 view) OR attitude* OR feeling* OR acceptance* OR accepts OR perception* OR misperception*).ab,ti.) NOT (news OR book* OR chapter* OR dissertation abstract*).pt.

LILACS

(smokefree OR smoking OR smoke) AND (opinions OR views OR attitudes)

Scientific Electronic Library Online [SciELO])

(smokefree OR smoking OR smoke) AND (opinions OR views OR attitudes)

WHO Global Health Library

(smokefree OR smoking OR smoke) AND (opinions OR views OR attitudes)

For peer review only

PRISMA-P (Preferred Reporting Items for Systematic review and Meta-Analysis Protocols) 2015 checklist: recommended items to address in a systematic review protocol*

Section and topic	Item No	Checklist item	Page
**ADMINISTRATIVE INFORMATION**			
Title:			
Identification	1a	Identify the report as a protocol of a systematic review	1
Update	1b	If the protocol is for an update of a previous systematic review, identify as such	N.A.
Registration	2	If registered, provide the name of the registry (such as PROSPERO) and registration number	2
Authors:			
Contact	3a	Provide name, institutional affiliation, e-mail address of all protocol authors; provide physical mailing address of corresponding author	1
Contributions	3b	Describe contributions of protocol authors and identify the guarantor of the review	10
Amendments	4	If the protocol represents an amendment of a previously completed or published protocol, identify as such and list changes; otherwise, state plan for documenting important protocol amendments	N.A.
Support:			
Sources	5a	Indicate sources of financial or other support for the review	10
Sponsor	5b	Provide name for the review funder and/or sponsor	10
Role of sponsor or funder	5c	Describe roles of funder(s), sponsor(s), and/or institution(s), if any, in developing the protocol	10
**INTRODUCTION**			
Rationale	6	Describe the rationale for the review in the context of what is already known	3
Objectives	7	Provide an explicit statement of the question(s) the review will address with reference to participants, interventions, comparators, and outcomes (PICO)	3
**METHODS**			
Eligibility criteria	8	Specify the study characteristics (such as PICO, study design, setting, time frame) and report characteristics (such as years considered, language, publication status) to be used as criteria for eligibility for the review	4
Information sources	9	Describe all intended information sources (such as electronic databases, contact with study authors, trial registers or other grey literature sources) with planned dates of coverage	5
Search strategy	10	Present draft of search strategy to be used for at least one electronic database, including planned limits, such that it could be repeated	Appendix I

For peer review only

Study records:			
Data management	11a	Describe the mechanism(s) that will be used to manage records and data throughout the review	5
Selection process	11b	State the process that will be used for selecting studies (such as two independent reviewers) through each phase of the review (that is, screening, eligibility and inclusion in meta-analysis)	6
Data collection process	11c	Describe planned method of extracting data from reports (such as piloting forms, done independently, in duplicate), any processes for obtaining and confirming data from investigators	6
Data items	12	List and define all variables for which data will be sought (such as PICO items, funding sources), any pre-planned data assumptions and simplifications	6
Outcomes and prioritization	13	List and define all outcomes for which data will be sought, including prioritization of main and additional outcomes, with rationale	7
Risk of bias in individual studies	14	Describe anticipated methods for assessing risk of bias of individual studies, including whether this will be done at the outcome or study level, or both; state how this information will be used in data synthesis	7
Data synthesis	15a	Describe criteria under which study data will be quantitatively synthesised	7
	15b	If data are appropriate for quantitative synthesis, describe planned summary measures, methods of handling data and methods of combining data from studies, including any planned exploration of consistency (such as I^2, Kendall's τ)	7
	15c	Describe any proposed additional analyses (such as sensitivity or subgroup analyses, meta-regression)	9
	15d	If quantitative synthesis is not appropriate, describe the type of summary planned	N.A.
Meta-bias(es)	16	Specify any planned assessment of meta-bias(es) (such as publication bias across studies, selective reporting within studies)	10
Confidence in cumulative evidence	17	Describe how the strength of the body of evidence will be assessed (such as GRADE)	-

*** It is strongly recommended that this checklist be read in conjunction with the PRISMA-P Explanation and Elaboration (cite when available) for important clarification on the items. Amendments to a review protocol should be tracked and dated. The copyright for PRISMA-P (including checklist) is held by the PRISMA-P Group and is distributed under a Creative Commons Attribution Licence 4.0.**

From: Shamseer L, Moher D, Clarke M, Ghersi D, Liberati A, Petticrew M, Shekelle P, Stewart L, PRISMA-P Group. Preferred reporting items for systematic review and meta-analysis protocols (PRISMA-P) 2015: elaboration and explanation. BMJ. 2015 Jan 2;349(jan02 1):g7647.

For peer review only
